# Exploring the potential mechanism of artemisinin against gastric cancer based on network pharmacology, machine learning and experimental validation

**DOI:** 10.3389/fonc.2026.1931227

**Published:** 2026-10-27

**Authors:** Chenchen Hu, Yu Zhao, Minghui Yun, Yu Shen, Weimin Lian, Bin Liu, Yong Li, Changhui Deng, Bowen Hou, Jiageng He

**Affiliations:** 1 The First Affiliated Hospital of Shihezi University, National Health Commission (NHC) Key Laboratory of Prevention and Treatment of Central Asia High Incidence Diseases (Co-construction), Shihezi, China; 2 Department of Gastrointestinal Surgery, The First Affiliated Hospital of Shihezi University, Shihezi, China; 3 Shihezi University School of Medicine, Shihezi, China; 4 Shihezi People’s Hospital (The Third Affiliated Hospital of Shihezi University School of Medicine), Shihezi, China

**Keywords:** artemisinin, ERK/MAPK, gastric cancer, machine learning, network pharmacology, *TOP2A*

## Abstract

**Background:**

Artemisinin, a natural active component extracted from *Artemisia annua*, exhibits potential anti-gastric cancer activity; however, its specific targets and molecular mechanisms remain unclear.

**Methods:**

Network pharmacology was used to predict the potential targets of artemisinin. Differential expression analysis and weighted gene co-expression network analysis (WGCNA) were performed to identify gastric cancer-related genes. Candidate targets of artemisinin against gastric cancer were obtained by intersecting the above datasets. Three machine learning algorithms, namely random forest, LASSO regression, and SVM-RFE, were further employed to screen hub targets, followed by molecular docking to evaluate the binding affinity between artemisinin and the hub targets. Finally, anti-gastric cancer effects and mechanisms were validated by *in vitro* experiments (CCK-8, EdU, colony formation, Transwell and Western blot).

**Results:**

A total of 27 candidate targets of artemisinin against gastric cancer were identified. Cross-screening by the three machine learning algorithms ultimately identified *TOP2A*, *CCNB2*, and *CCNA2* as hub targets. Molecular docking revealed low binding energies (all below −5.0 kcal/mol) between artemisinin and these three targets, suggesting potential binding affinity. *In vitro* experiments demonstrated that artemisinin significantly downregulated the mRNA expression levels of the three targets and suppressed the proliferation, migration, and invasion of gastric cancer cells. Mechanistic investigation showed that artemisinin treatment decreased p-ERK levels, and the anti-gastric cancer effects of artemisinin were partially reversed by the ERK pathway activator C16-PAF.

**Conclusions:**

*In vitro*, artemisinin suppresses ERK/MAPK pathway activation and reduces the mRNA expression of cell cycle-related *TOP2A*, *CCNB2* and *CCNA2*, leading to inhibited proliferation, migration and invasion of gastric cancer cells.

## Introduction

Gastric cancer (GC) is the fifth most common malignancy and the third leading cause of cancer-related death worldwide (1, 2). Although the incidence has declined in some regions due to improved public health measures and dietary changes, the annual number of new GC cases is projected to reach 1.1 million globally by 2030 (3). Gastric adenocarcinoma accounts for the majority of GC cases and is often diagnosed at an advanced stage due to its asymptomatic nature in early phases, which significantly contributes to the high mortality rate, with a 5-year survival rate of less than 30% for advanced GC (4). Although surgical techniques, chemotherapy, and targeted therapy have improved the clinical management of GC, these approaches still face limitations such as high toxicity, drug resistance, and limited efficacy in advanced disease. Therefore, there is an urgent need for effective alternative or adjunctive therapeutic strategies.

Traditional Chinese medicine (TCM) has been used in China for thousands of years and remains an important source for modern drug discovery. Many clinically used anticancer agents, such as paclitaxel, camptothecin and their derivatives, were originally derived from medicinal plants (5). Artemisinin, a potent natural compound extracted from *Artemisia annua* L., is a representative example of TCM-inspired drug discovery; it was originally used as an antimalarial agent and has recently been found to possess antitumor activity. Multiple studies have shown that artemisinin and its derivatives can inhibit gastric cancer cell proliferation, reduce tumor volume, and induce cell cycle arrest in preclinical models (6, 7). Furthermore, artemisinin has been reported to suppress epithelial-mesenchymal transition (EMT) and metastasis of gastric cancer cells by regulating signaling pathways such as PI3K/Akt and MAPK (8, 9). These findings indicate that artemisinin has the potential to inhibit both tumor growth and metastasis; however, its specific molecular mechanisms remain unclear. Given that conventional chemotherapy for gastric cancer is often associated with substantial toxicity and impaired quality of life (10), natural products such as artemisinin, which generally have favorable safety profiles, may serve as promising adjunctive therapeutic options. Identifying the molecular targets of artemisinin in gastric cancer, as attempted in this study, may contribute to the development of low-toxicity therapeutic strategies that could reduce treatment-related adverse effects and improve patients’ quality of life. In this study, network pharmacology was employed to predict the potential targets of artemisinin (11). Machine learning algorithms, including random forest, LASSO regression, and SVM-RFE, were used to screen key hub genes from the candidate targets, and molecular docking was performed to evaluate the binding affinity between artemisinin and the target proteins (12). Finally, the predicted results were validated through *in vitro* experiments.

## Materials and methods

### Prediction of artemisinin targets

Artemisinin-related targets were retrieved from four databases: HERB (http://herb.ac.cn/), PubChem (https://pubchem.ncbi.nlm.nih.gov/), Swiss Target Prediction (http://www.swisstargetprediction.ch/), and SuperPred (https://prediction.charite.de/). The PubChem database was used only to obtain the SMILES chemical structure of artemisinin and was not used for target prediction. Known pharmacological targets of artemisinin were collected from the HERB database. The SMILES structure of artemisinin was downloaded from PubChem and subsequently submitted to the Swiss Target Prediction and SuperPred databases to expand the target search. For each predicted target, the corresponding gene name was retrieved using the UniProt database (https://www.uniprot.org/). Finally, all identified targets were merged and deduplicated to generate the final list of artemisinin-related targets.

### Identification of gastric cancer-related genes

RNA sequencing data of gastric cancer were obtained from the UCSC Xena database (https://xena.ucsc.edu/), which integrates resources from TCGA, GTEx, ICGC and other databases, covering a wide range of biological samples and tissue types. A total of 624 RNA-seq samples (FPKM) were downloaded from UCSC Xena, including 414 tumor tissues and 210 normal tissues (derived from both GTEx and adjacent normal tissues from TCGA). Since the downloaded FPKM data had already undergone batch effect removal and log2 transformation, these steps were not repeated. In addition, the online tools UALCAN (https://ualcan.path.uab.edu/) and GEPIA (http://gepia.cancer-pku.cn/) were used to validate gene expression levels and perform prognostic analysis.

Differential expression analysis of the gastric cancer FPKM data was performed using the limma package (13). The p-values were adjusted for multiple testing using the Benjamini-Hochberg false discovery rate (FDR) method. Genes with adjusted p < 0.05 and |logFC| > 1 were considered differentially expressed genes (DEGs). Among these, genes with logFC > 1 were defined as upregulated, and those with logFC < -1 as downregulated.

WGCNA was employed to identify gene co-expression modules associated with gastric cancer (14). Outlier samples were first removed, and a soft threshold (β = 4) was selected based on the criterion of scale-free topology fit index R² > 0.85. An adjacency matrix was constructed using this soft threshold and then transformed into a topological overlap matrix (TOM). Co-expression modules were identified by hierarchical clustering. The correlation between each module and the gastric cancer phenotype was calculated, and the module with the highest correlation was selected as the key module. Finally, gastric cancer-related genes were identified by intersecting DEGs with the genes from the key module using a Venn diagram.

### Screening of potential targets of artemisinin against gastric cancer

Potential targets of artemisinin against gastric cancer were identified by intersecting the artemisinin-related pharmacological targets with gastric cancer-related genes.

### PPI network and enrichment analysis

To identify hub targets of artemisinin against gastric cancer, a protein-protein interaction (PPI) network of the potential targets was constructed using the STRING database (https://string-db.org/). The TSV format file was exported from STRING and imported into Cytoscape software (version 3.10.1) for network analysis and visualization (15). The CytoHubba plugin, which is widely used for hub gene identification in PPI networks, was employed (11). Three topological algorithms, namely MCC, MNC, and Degree, were applied, and the top 10 genes from each algorithm were selected. The intersection of the genes identified by the three algorithms was defined as candidate hub genes. To further explore the biological functions and signaling pathways of these hub genes, Gene Ontology (GO) biological process analysis and Kyoto Encyclopedia of Genes and Genomes (KEGG) pathway enrichment analysis were performed using the ClusterProfiler package (16, 17). Visualization was carried out using the ggplot2 and igraph packages. Terms with p < 0.05 and q < 0.2 were considered significantly enriched.

### Machine learning for screening hub targets

To further prioritize candidate genes that are closely associated with the tumor phenotype, tumor tissues were labeled as 1 and normal tissues as 0. Three machine learning algorithms, namely random forest, LASSO regression, and SVM-RFE, were applied to screen candidate hub genes. These three algorithms were selected because they are based on different mathematical principles: random forest excels at capturing nonlinear relationships, LASSO regression is advantageous for sparse feature selection, and SVM-RFE performs robustly in high-dimensional data analysis. The intersection of the three algorithms was expected to reduce false-positive and false-negative results that may arise from a single algorithm. These algorithms classify genes that discriminate tumor from normal tissues (i.e., tumor phenotype drivers), rather than genes that directly bind to artemisinin (i.e., drug-binding affinity). Therefore, the machine learning step was used only to prioritize candidate genes and did not by itself establish a drug–target relationship. The direct binding potential of artemisinin to these targets was subsequently evaluated by molecular docking, and the regulatory effects of artemisinin on their expression were validated by qRT-PCR and Western blot. First, random forest analysis was performed using the R package “randomForest” with default parameters (18). Candidate genes were ranked according to their importance, and the top 5 genes based on the mean decrease in Gini index were selected as key candidate hub genes. Second, LASSO regression analysis was conducted using the “glmnet” package (19). The optimal lambda value that minimized classification error was identified to select variables for constructing the best classification model. Finally, the SVM-RFE method was used to determine the importance and ranking of each gene (20). Five-fold cross-validation was performed to obtain the error rate and accuracy for each iteration combination. The gene set with the highest accuracy and lowest error rate was selected as candidate key genes. Based on these analyses, the intersection of genes identified by the three machine learning algorithms was defined as the hub targets. These hub targets represent tumor phenotype drivers that may be involved in gastric cancer progression, and their potential as artemisinin-binding targets was further evaluated by molecular docking and *in vitro* experiments.

### Molecular docking

In this study, molecular docking was performed on the hub targets. The chemical structure of artemisinin was obtained from the PubChem database. The crystal structure of TOP2A was retrieved from the RCSB Protein Data Bank (PDB) database (https://www.rcsb.org/; PDB ID: 1ZXM). Since no experimentally determined crystal structures were available for CCNB2 and CCNA2, their AlphaFold-predicted structures (UniProt ID: O95067 for CCNB2 and P20248 for CCNA2) were used instead. Molecular docking was carried out using AutoDock Tools (version 1.5.7) (21). The docking results were analyzed based on the binding affinity (kcal/mol) and the root-mean-square deviation (RMSD) relative to the best-scoring conformation. The complexes with the highest binding affinity and lowest energy were compared and saved in pdbqt format. Finally, visualization of the compound-protein binding modes was performed using Discovery Studio 4.5. Molecular docking is a theoretical prediction and does not directly demonstrate physical binding between artemisinin and the target proteins.

### Cell culture

The human gastric cancer cell lines SGC7901 and SNU-5 were obtained from Cyagen Biosciences. The cells were cultured in RPMI 1640 medium supplemented with 10% fetal bovine serum and 1% penicillin-streptomycin. All cell lines were confirmed to be mycoplasma-free and maintained in a humidified incubator at 37 °C with 5% CO_2_.

### RNA extraction and quantitative real-time PCR

The qRT-PCR primers used in this study were designed and synthesized by Shanghai GenePharma Co., Ltd. The primer sequences were as follows:

GAPDH: Forward 5′- AAGCTCATTTCCTGGTATGACA -3′,
Reverse 5′- GGAGATGCTCAGTGTTGGGG -3′;
*CCNB2*: Forward 5′- AGTTACAACCAGAGCAGCACA -3′,
Reverse 5′- GGACCCTTTGGAGCCAACTT -3′;
*CCNA2*: Forward 5′- CTCCAAGAGGACCAGGAGAAT -3′,
Reverse 5′- TTTCCAAGGAGGAACGGTGA -3′;
*TOP2A*: Forward 5′- TCCCAAACTCGATGATGCCA -3′,
Reverse 5′- AACCACACCAAGGCCTGAAA -3′.

Total RNA was extracted from the two cell lines using the E.Z.N.A. Total RNA Kit I (Omega Bio-Tek) according to the manufacturer’s instructions. Complementary DNA (cDNA) was synthesized using the RevertAid First Strand cDNA Synthesis Kit (Thermo Fisher Scientific). Quantitative real-time PCR (qRT-PCR) was performed on the CFX96 Touch Real-Time PCR Detection System (Bio-Rad). GAPDH was used as an internal control, and each experiment was performed in triplicate.

### Western blot analysis

Total protein was extracted from cells or tissues using a total protein extraction kit (Sangon Biotech Co., Ltd., Shanghai, China) according to the manufacturer’s instructions. Protein samples were separated by SDS-PAGE at 80 V for 3 hours and then transferred onto polyvinylidene difluoride (PVDF) membranes at 80 V for 1 hour. The membranes were blocked with a commercial blocking buffer (Servicebio, Wuhan, China) at room temperature for 2 hours and then incubated with primary antibodies at 4 °C for 14–16 hours. After washing, the membranes were incubated with secondary antibodies at the same temperature for 2 hours. Between each step, the membranes were washed three times with 1× TBST buffer for 10 minutes each to avoid reagent contamination. The immunoreactive bands were visualized using a chemiluminescent substrate prepared according to the manufacturer’s instructions, and images were captured using a gel imaging system in a dark chamber.

The following primary antibodies were used: Ki-67 (BOSTER, PB9026, 1:1000), Vimentin (Abcam, ab92547, 1:1000), p-ERK (Cell Signaling Technology, 4370, 1:1000), ERK (Cell Signaling Technology, 4695, 1:1000), MMP2 (BOSTER, BM4075, 1:1000), MMP9 (BOSTER, BM4089, 1:1000), and GAPDH (ZSGB-BIO, TA-08, 1:1000).

### Cell proliferation assay

Cell proliferation was assessed using CCK-8, EdU, and colony formation assays according to the manufacturers’ instructions. All CCK-8 experiments were performed in biological triplicate (three independent experiments). The percentage of cell viability was calculated relative to the 0 μM untreated control group. Statistical analysis and data visualization were performed using GraphPad Prism 8.0 software.

### Transwell assay

Cell migration assay was performed using 24-well Transwell chambers with polycarbonate membranes of 8.0 μm pore size. Gastric cancer cells were seeded into the upper chamber in serum-free DMEM medium, while the lower chamber was filled with RPMI 1640 medium containing 10% fetal bovine serum. After 24 hours, the membranes were fixed with 4% paraformaldehyde at 4 °C for 15 minutes and then stained with 0.1% crystal violet for 20 minutes. The stained cells were photographed and counted. The cell invasion assay was performed similarly, except that the membranes were pre-coated with Matrigel before cell seeding.

### Statistical analysis

All data in this study were obtained from three independent experiments. Statistical analysis was performed using GraphPad Prism 8.0 software. Comparisons between two groups were conducted using unpaired Student’s t-test, while comparisons among multiple groups were performed using one-way analysis of variance (ANOVA). A P-value ≥ 0.05 was considered not statistically significant and denoted as “ns”. P-values < 0.05, < 0.01, and < 0.001 were considered statistically significant and denoted as *, **, and ***, respectively.

## Results

### Identification of artemisinin targets and gastric cancer-related genes

Artemisinin targets were obtained by searching the HERB, Swiss Target Prediction, and SuperPred databases. After ID conversion using the UniProt database and removal of duplicates, a total of 550 artemisinin-related targets were identified.

To identify gastric cancer-related genes, differential expression analysis and WGCNA were performed. As shown in **Figure 1A**, a total of 2,892 differentially expressed genes (DEGs) were identified using the limma package, including 1,371 upregulated and 1,521 downregulated genes. The top 5,000 genes ranked by median absolute deviation were selected for WGCNA analysis. After sample clustering, two outlier samples were excluded (**Supplementary Figure S1**). As shown in **Supplementary Figure S2**, based on the criterion of scale-free topology fit index R² > 0.85, the optimal soft threshold was determined to be β = 4, at which the network maintained a high mean connectivity, ensuring the stability and reliability of subsequent module division. A co-expression network of gastric cancer-related genes was constructed using this soft threshold, and a total of 11 independent co-expression modules were identified by the dynamic tree-cutting method (**Supplementary Figure S3**). Module–trait association analysis is shown in **Figure 1B**. Among these modules, the blue module, which contained 1,201 genes, showed the highest correlation with gastric cancer (r = 0.91, p < 0.001), indicating that genes in this module are closely associated with the development and progression of gastric cancer. Therefore, the blue module was selected as the key module. By intersecting the genes in this module with the DEGs, a total of 507 gastric cancer-related genes were obtained. By intersecting the 507 gastric cancer-related genes with the 550 artemisinin-related targets, 27 common targets were obtained (**Figure 1C**). These were considered candidate key targets of artemisinin against gastric cancer and were used for subsequent network pharmacology analysis.

**Figure 1:**
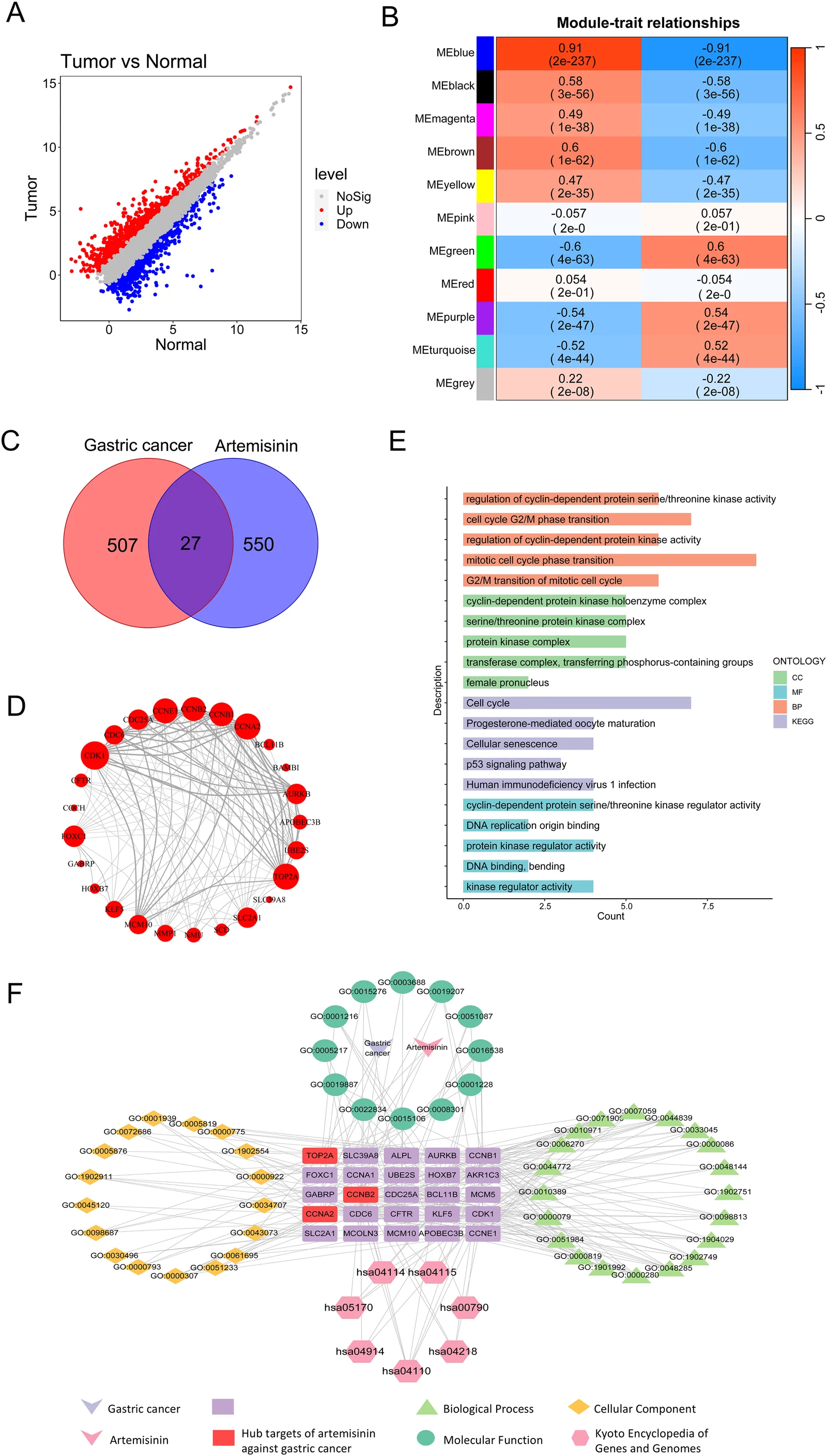
In silico screening and functional enrichment analysis of artemisinin targets against gastric cancer. **(A)** Volcano plot of differentially expressed genes (DEGs) between gastric cancer and normal tissues from the TCGA-STAD dataset (|logFC| > 1, adjusted *p* < 0.05). **(B)** WGCNA module-trait correlation heatmap showing the blue module as the most significantly correlated with gastric cancer (r = 0.91, *p* < 0.001). **(C)** Venn diagram showing the intersection of gastric cancer-related targets and artemisinin targets. **(D)** PPI network of 27 potential targets. **(E)** GO and KEGG enrichment analysis of the 27 potential targets. **(F)** Disease-compound-target-pathway network.

### GO and KEGG enrichment analysis of potential targets of artemisinin against gastric cancer

GO analysis revealed that the biological processes of the potential targets of artemisinin against gastric cancer were significantly enriched in regulation of cyclin-dependent protein serine/threonine kinase activity, cell cycle G2/M phase transition, and regulation of cyclin-dependent protein kinase activity. Regarding cellular components, the potential targets were mainly localized in the cyclin-dependent protein kinase holoenzyme complex, serine/threonine protein kinase complex, and protein kinase complex. For molecular functions, the potential targets were enriched in cyclin-dependent protein serine/threonine kinase regulator activity, DNA replication origin binding, and protein kinase regulator activity. Furthermore, KEGG pathway enrichment analysis showed that the potential targets were significantly enriched in pathways including Cell cycle, Progesterone-mediated oocyte maturation, Cellular senescence, and the p53 signaling pathway (**Figure 1E**).

Collectively, the core targets were mainly enriched in biological processes such as cell cycle G2/M phase transition and regulation of CDK kinase activity, as well as pathways including the Cell cycle and p53 signaling pathway, suggesting that artemisinin may exert its anti-gastric cancer effect by regulating the cell cycle process.

### Machine learning for hub target identification

To further screen core targets from the 27 candidate targets, a PPI network was first constructed with an interaction confidence score of 0.15, and isolated nodes were hidden (**Figure 1D**). The CytoHubba plugin was used to calculate the importance of each node using three topological algorithms: MCC, MNC, and Degree. The top 10 targets from each algorithm were selected, and their intersection yielded 7 candidate hub genes, including *CCNA2*, *CCNB1*, *CCNB2*, *CCNE1*, *CDC25A*, *CDK1*, and *TOP2A*.

To further refine the hub targets, three machine learning algorithms were applied. First, random forest analysis was performed, and the top 5 important features based on the Gini index were selected as candidate hub targets (**Supplementary Figures S4**, **S5**). Second, an optimized model was constructed using LASSO regression, from which 3 candidate hub targets were identified (**Supplementary Figures S6**, **S7**). Finally, SVM-RFE analysis showed that the model achieved the lowest error rate and the highest accuracy when 6 features were included (**Supplementary Figures S8**, **S9**).

The intersection of the results from the three machine learning algorithms ultimately identified *TOP2A*, CCNB2, and *CCNA2* as the hub targets of artemisinin against gastric cancer (**Table 1**). **Table 1** summarizes the candidate hub targets obtained by the CytoHubba algorithm and the three machine learning algorithms.

**Table 1:** Screening of hub targets by CytoHubba algorithms and machine learning methods.

Analysis type	Method	Candidate target
CytoHubba	MCC	CDK1, *TOP2A*, *CCNA2*, CCNB1, *CCNB2*, CCNE1, CDC25A, AURKB, CDC6, MCM10
	MNC	CDK1, *CCNA2*, *TOP2A*, CCNE1, *CCNB2*, CCNB1, CDC25A, AURKB, FOXC1, CDC6
	Degree	CDK1, *CCNA2*, *TOP2A*, CCNE1, *CCNB2*, CCNB1, FOXC1, CDC25A, SLC2A1, AURKB
Machine learning	RF	*TOP2A*, *CCNB2*, CDK1, *CCNA2*, CCNB1
	LASSO	*CCNA2*, *CCNB2*, *TOP2A*
	SVM-RFE	*TOP2A*, CCNB1, *CCNB2*, *CCNA2*, CDK1, CCNE1

MCC, maximal clique centrality; MNC, maximum neighborhood component; Degree, node connectivity degree; RF, random forest; SVM-RFE, support vector machine-recursive feature elimination.

### Construction of the “disease–compound–target–pathway” network

A “disease–compound–target–pathway” network was constructed using Cytoscape software to visualize the associations among gastric cancer, artemisinin, the 27 potential targets, and 7 key KEGG pathways (**Figure 1F**). The network consisted of one compound node (artemisinin), one disease node (gastric cancer), 27 target nodes, and 7 pathway nodes. In this network, the three hub targets *TOP2A*, CCNB2, and *CCNA2* occupied central positions, with degree values of 12, 24, and 18, respectively, further suggesting that they are key nodes mediating the anti-gastric cancer effect of artemisinin.

### Validation of hub target expression in gastric cancer

The mRNA expression levels of the three hub targets in gastric cancer and normal tissues were analyzed using the UALCAN database. As shown in **Figure 2A**, the expression levels of *TOP2A*, CCNB2, and *CCNA2* were significantly higher in gastric cancer tissues than in normal gastric tissues. To further validate this result, the expression of these genes in stomach adenocarcinoma (STAD) was analyzed based on the TCGA database, which also showed significantly high expression in tumor tissues (**Figure 2B**).

**Figure 2:**
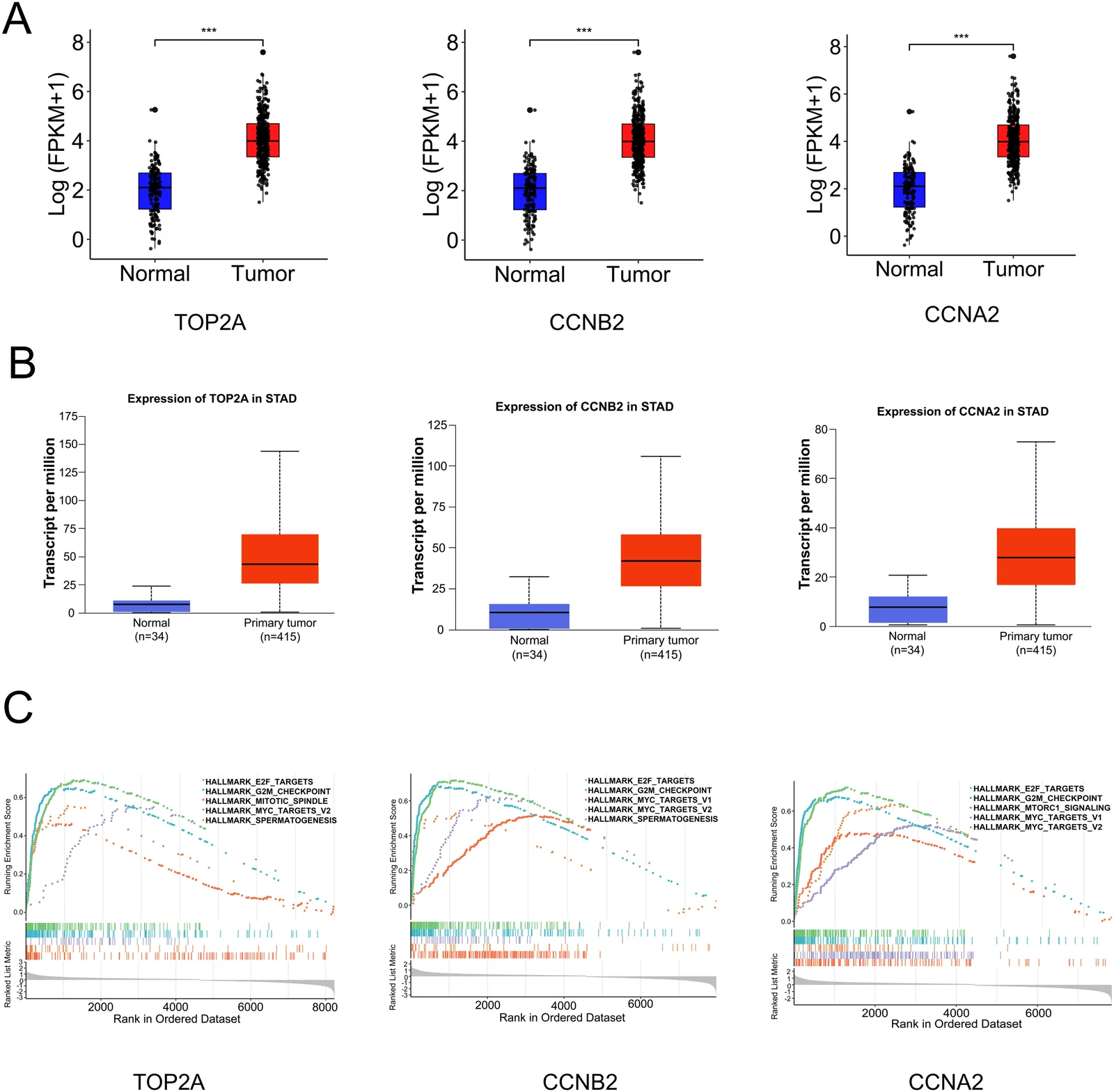
Validation of hub target expression and pathway enrichment in gastric cancer. **(A)** mRNA expression levels of TOP2A, CCNB2, and CCNA2 in gastric cancer versus normal tissues from the UALCAN database. **(B)** Validation of mRNA expression in stomach adenocarcinoma (STAD) versus normal tissues from the TCGA database. **(C)** GSEA revealed enrichment of cell cycle pathways (E2F_TARGETS and G2M_CHECKPOINT) in the high-expression group of hub targets. Data are presented as mean ± SD. ***p < 0.001, **p < 0.01, *p < 0.05 compared with the normal group.

The protein expression levels of the three hub targets in gastric cancer tissues were analyzed using the Human Protein Atlas (HPA) database (https://www.proteinatlas.org/). As shown in **Supplementary Figure S10**, *TOP2A*, *CCNB2*, and *CCNA2* exhibited high expression levels in gastric adenocarcinoma tissues across different stages.

Although *TOP2A*, *CCNB2*, and *CCNA2* were all significantly highly expressed in gastric cancer tissues, Kaplan–Meier survival analysis showed no significant statistical association between their expression levels and the overall survival (OS) of gastric cancer patients (**Supplementary Figure S11**). This result suggests that these cell cycle-related genes may primarily participate in the development and malignant phenotype regulation of gastric cancer rather than serving as independent prognostic predictors for patients with advanced disease. Their clinical prognostic value warrants further validation in larger cohort studies.

### GSEA analysis of hub target genes

To further explore the potential mechanisms of the hub target genes in gastric cancer, samples from the gastric cancer dataset were divided into high-expression and low-expression groups based on the median expression level of each hub gene. Gene Set Enrichment Analysis (GSEA) was performed to evaluate enrichment differences between the two groups. The reference gene set h.all.v2024.1.Hs.symbols.gmt was used for the analysis. The results showed that the high-expression groups of *TOP2A*, *CCNA2*, and *CCNB2* were significantly enriched in multiple cell cycle-related pathways, such as HALLMARK_E2F_TARGETS and HALLMARK_G2M_CHECKPOINT, suggesting that these genes may participate in the development and progression of gastric cancer by promoting cell cycle progression. In addition, pathways related to cell proliferation, mitosis, and tumor metabolism, including HALLMARK_MITOTIC_SPINDLE, HALLMARK_MYC_TARGETS_V1/V2, and HALLMARK_MTORC1_SIGNALING, were also significantly enriched in the high-expression groups (**Figure 2C**). These results indicate that high expression of *TOP2A*, *CCNA2*, and *CCNB2* is closely linked to gastric cancer progression, which may facilitate malignant development via modulating cell cycle progression and cell proliferation.

### Immune cell infiltration analysis

Based on the TIMER2.0 database (http://timer.cistrome.org/), the correlations between the expression of the three hub targets (*TOP2A*, *CCNB2*, and *CCNA2*) and the infiltration levels of six major immune cell types (B cells, CD4+ T cells, CD8+ T cells, neutrophils, macrophages, and dendritic cells) were evaluated. The analysis results showed that the three hub targets were significantly negatively correlated with most immune cell types, particularly CD4+ T cells, CD8+ T cells, and macrophages (**Figure 3**). These findings suggest a potential link between hub target expression and the gastric cancer immune microenvironment, though the underlying regulatory mechanisms require further experimental validation.

**Figure 3:**
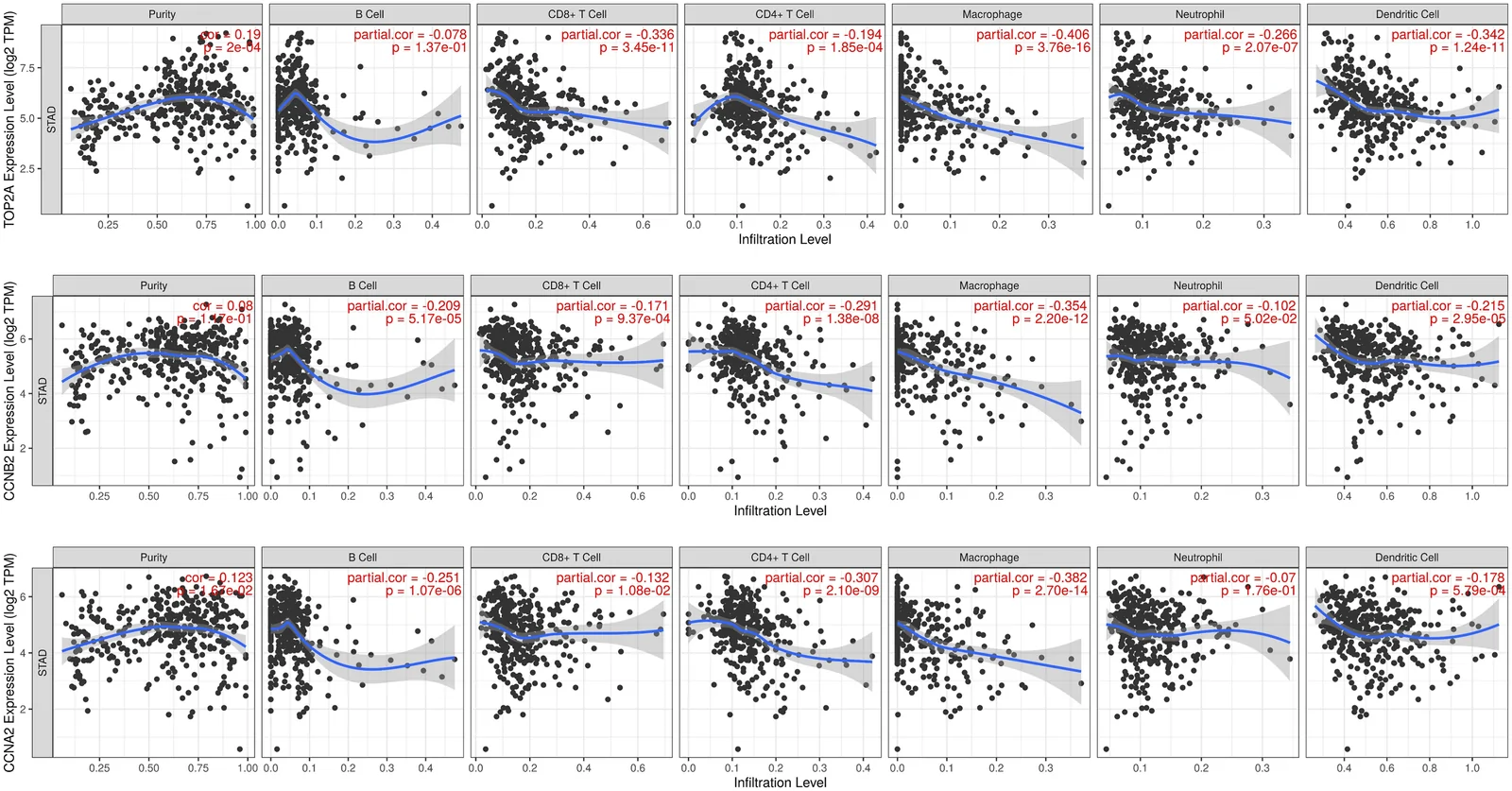
Correlation between hub gene expression and immune cell infiltration in gastric cancer. Scatter plots showing the correlation between the expression of TOP2A, CCNB2, and CCNA2 and the infiltration levels of six immune cell types: B cells, CD4+ T cells, CD8+ T cells, macrophages, neutrophils, and dendritic cells (columns 1–7, respectively). Data were obtained from the TIMER database. Each dot represents an individual sample. Rho and p-values are shown for each correlation.

### Molecular docking validation

In molecular docking, a lower binding energy indicates a more stable interaction between the ligand and receptor, and a binding energy below −5.0 kcal/mol is generally considered to suggest potential binding affinity. In this study, molecular docking was performed between artemisinin and the three hub targets (*TOP2A*, *CCNB2*, and *CCNA2*). As shown in **Table 2**, the binding energies of artemisinin with the three hub targets were all below −5.0 kcal/mol (*TOP2A*: −6.83 kcal/mol; *CCNB2*: −7.30 kcal/mol; *CCNA2*: −6.87 kcal/mol), suggesting potential binding affinity between artemisinin and these target proteins.

**Table 2:** Molecular docking results of artemisinin with the core hub targets.

Target	PDB ID/source	Binding energy (kcal/mol)
CCNA2	AlphaFold (UniProt: P20248)	-6.87
CCNB2	AlphaFold (UniProt: O95067)	-7.30
TOP2A	1ZXM	-6.83

Comparison with reference compounds (etoposide for TOP2A, salicylic acid for CCNA2, and seliciclib for CCNB2) is provided in the Discussion section. Binding mode details are shown in **Figure 4**.

Binding mode analysis (**Figure 4**) showed that artemisinin formed stable interactions with *CCNA2* (**Figure 4A**), *CCNB2* (**Figure 4B**), and *TOP2A* (**Figure 4C**) through hydrogen bonds, hydrophobic interactions, and π-alkyl interactions. The specific residues involved in these interactions are summarized in **Table 3**.

**Figure 4:**
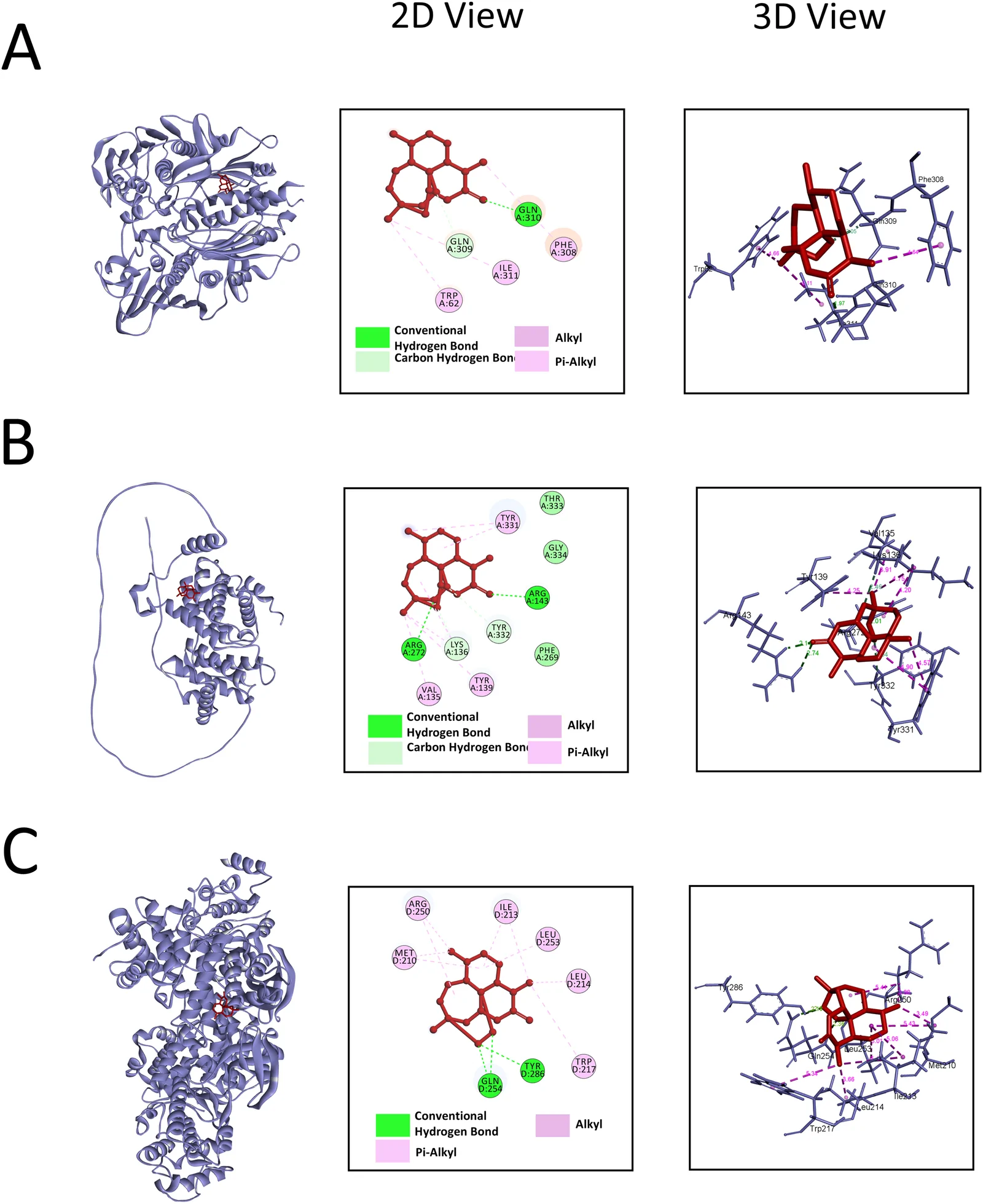
Molecular docking analysis of artemisinin with the three hub targets. **(A)** Artemisinin binding with CCNA2 (AlphaFold-predicted structure, UniProt ID: P20248). From left to right: overall structure showing binding site, 2D interaction diagram, and 3D binding mode. **(B)** Artemisinin binding with CCNB2 (AlphaFold-predicted structure, UniProt ID: O95067). From left to right: overall structure, 2D interaction diagram, and 3D binding mode. **(C)** Artemisinin binding with TOP2A (PDB: 1ZXM). From left to right: overall structure, 2D interaction diagram, and 3D binding mode.

**Table 3:** Interacting residues of artemisinin with the three hub targets identified by molecular docking.

Target	Hydrogen bonds (residues)	Hydrophobic contacts	π-alkyl interactions
CCNA2	GLN A:309	PHE A:308; ILE A:311	TRP A:62
CCNB2	ARG A:74; LYS A:136; ASN A:143	VAL A:135; TYR A:139; PHE A:269	TYR A:331; TYR A:332
TOP2A	GLU D:256; LYS D:228	ARG D:250; ILE D:213; LEU D:253; LEU D:214; MET D:210; TRP D:217	_

Molecular docking is a stochastic process, and the results may vary slightly between independent runs. The results shown here are from the lowest-energy binding conformation.

### Artemisinin downregulates the expression of hub targets

To validate the roles of the hub targets (*TOP2A*, *CCNB2*, and *CCNA2*) identified by network pharmacology and machine learning in the anti-gastric cancer effect of artemisinin, a series of *in vitro* experiments were performed.

Based on the results of preliminary concentration-gradient screening experiments, artemisinin was used at concentrations of 150 μM for SGC7901 cells and 200 μM for SNU-5 cells in subsequent functional assays. These concentrations yielded substantial inhibitory effects on gastric cancer cell viability without triggering complete cell death, enabling phenotypic evaluation of cell proliferation, migration, and invasion. Of note, the anti-tumor activity of artemisinin and its derivatives is dose-dependent and varies greatly among different tumor cell lines. Gravett et al. reported that artemisone and artemisinin caused dose-dependent decreases in cancer cell number, with their anti-proliferative effects varying considerably among a panel of human cancer cell lines (22). These findings support the relatively high concentrations used in our study. The dose-response tendency is presented in **Figure 5C** and **Supplementary Figure S12**.

**Figure 5:**
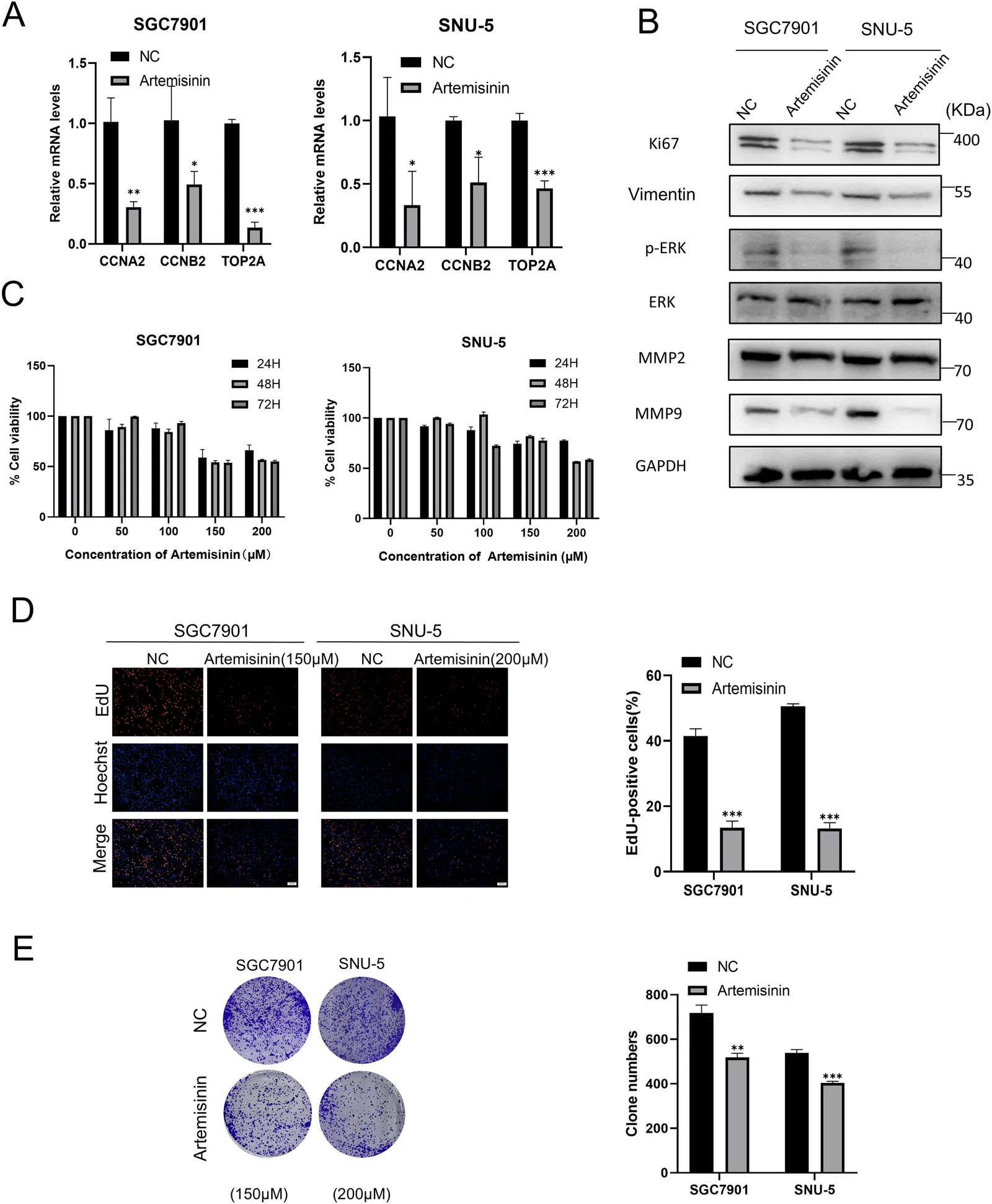
Artemisinin downregulates hub target expression and suppresses proliferation of gastric cancer cells. **(A)** qRT-PCR analysis of TOP2A, CCNB2 and CCNA2 mRNA levels in SGC7901 and SNU-5 cells after artemisinin treatment. **(B)** Western blot analysis of proliferation, migration and ERK pathway-related proteins. **(C)** CCK-8 assay showing cell viability of SGC7901 and SNU-5 cells after artemisinin treatment for 24, 48, and 72 h. All experiments were conducted in biological triplicate (three independent experiments). Data are presented as mean ± SD. **(D)** EdU staining assay with representative fluorescence images and quantification of EdU-positive cells. **(E)** Colony formation assay with representative images and quantification of colony numbers. Data are presented as mean ± SD from three independent experiments. ***p < 0.001, **p < 0.01, *p < 0.05 compared with the control group.

The effect of artemisinin on the mRNA expression of the key targets in gastric cancer cells was examined by qRT-PCR. The results showed that compared with the control group, artemisinin treatment significantly downregulated the mRNA expression levels of *TOP2A*, *CCNB2*, and *CCNA2* in both cell lines (**Figure 5A**).

Western blot analysis was performed to detect the effect of artemisinin on proliferation- and migration-related proteins. The results showed that artemisinin treatment significantly inhibited the expression of the proliferation marker Ki-67, migration-related proteins (Vimentin, MMP2, MMP9), and phosphorylated ERK (p-ERK), while total ERK protein levels showed no significant change (**Figure 5B**).

### Artemisinin suppresses the proliferative capacity of gastric cancer cells

To functionally validate the anti-tumor effect of artemisinin, the CCK-8 assay, EdU staining, and colony formation assay were used to evaluate its impact on the proliferative capacity of gastric cancer cells.

The CCK-8 assay (**Figure 5C**) showed that artemisinin inhibited the viability of SGC7901 and SNU-5 cells in a time- and dose-dependent manner. Cell viability gradually decreased with increasing drug concentration and treatment duration, and was markedly suppressed at concentrations of 150 μM (SGC7901) and 200 μM (SNU-5). The dose-response cytotoxicity curves of artemisinin in SGC7901 and SNU-5 cells are shown in **Supplementary Figure S12**.

The EdU staining assay (**Figure 5D**) further demonstrated that treatment with 150 μM artemisinin in SGC7901 cells and 200 μM artemisinin in SNU-5 cells significantly reduced the percentage of EdU-positive cells, indicating that artemisinin effectively inhibits DNA synthesis and proliferative activity in gastric cancer cells.

The colony formation assay (**Figure 5E**) showed that artemisinin treatment significantly reduced both the size and number of colonies formed by SGC7901 and SNU-5 cells. Collectively, these results demonstrate that artemisinin effectively suppresses the *in vitro* proliferation and clonogenicity of gastric cancer cells.

### Artemisinin suppresses the migration and invasion abilities of gastric cancer cells

The effect of artemisinin on the migration and invasion abilities of gastric cancer cells was investigated using Transwell assays. As shown in **Figure 6A**, in the migration assay, the number of cells crossing the polycarbonate membrane was significantly reduced in SGC7901 cells treated with 150 μM artemisinin and SNU-5 cells treated with 200 μM artemisinin compared with the control group. The invasion assay results (**Figure 6B**) showed that in Matrigel-precoated Transwell chambers, the number of cells penetrating the Matrigel was also significantly lower in the artemisinin-treated groups than in the control group. These results indicate that artemisinin not only inhibits the proliferation of gastric cancer cells but also effectively attenuates their migration and invasion abilities.

**Figure 6:**
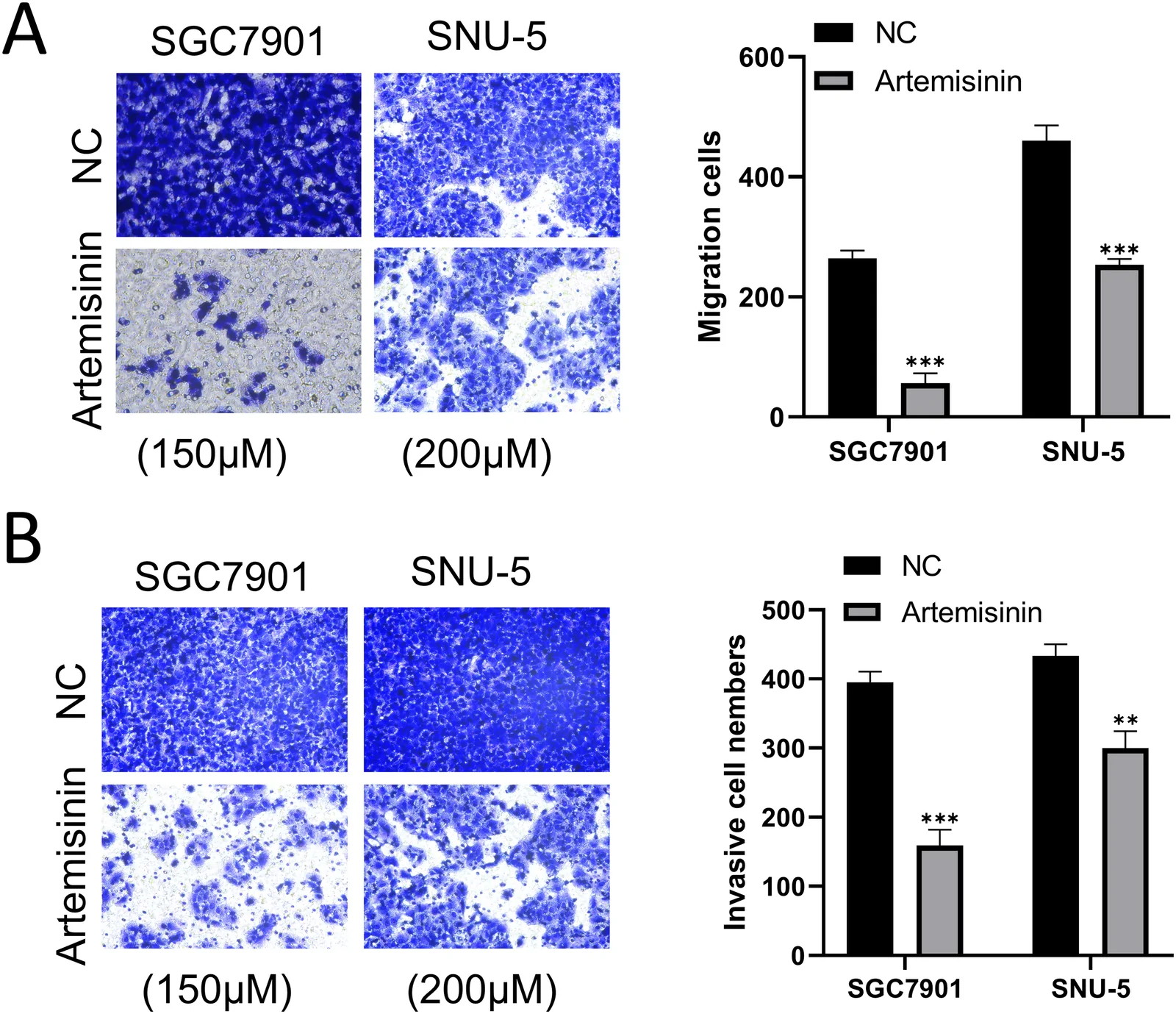
Artemisinin inhibits migration and invasion of gastric cancer cells. **(A)** Transwell migration assay. Representative images of migrated cells and quantitative analysis of migrated cell numbers. **(B)** Transwell invasion assay (Matrigel-coated). Representative images of invaded cells and quantitative analysis of invaded cell numbers. Data are presented as mean ± SD from three independent experiments. **p < 0.01, ***p < 0.001 compared with the control group.

### Artemisinin exerts anti-gastric cancer effects by regulating hub targets via the ERK/MAPK pathway

To investigate the molecular mechanism by which artemisinin inhibits the proliferation, migration, and invasion of gastric cancer cells, and considering the previously identified cell cycle-related hub targets *TOP2A*, *CCNA2*, and *CCNB2*, rescue experiments were performed using the ERK pathway activator C16-PAF to determine whether artemisinin exerts its anti-gastric cancer effects through the ERK/MAPK pathway.

The EdU staining results (**Figure 7A**) showed that compared with the control group, the percentage of EdU-positive cells was significantly decreased in SGC7901 and SNU-5 cells following artemisinin treatment alone. However, upon combined treatment with C16-PAF, the percentage of EdU-positive cells markedly increased, indicating that C16-PAF effectively reversed the artemisinin-induced proliferation inhibition. The colony formation assay further confirmed (**Figure 7B**) that artemisinin significantly reduced the number of colonies formed by both cell lines, whereas the addition of C16-PAF markedly restored the long-term clonogenic ability of the cells. These results suggest that the inhibitory effects of artemisinin on both short-term DNA synthesis and long-term proliferative potential in gastric cancer cells are dependent on the inhibition of the ERK/MAPK pathway.

**Figure 7:**
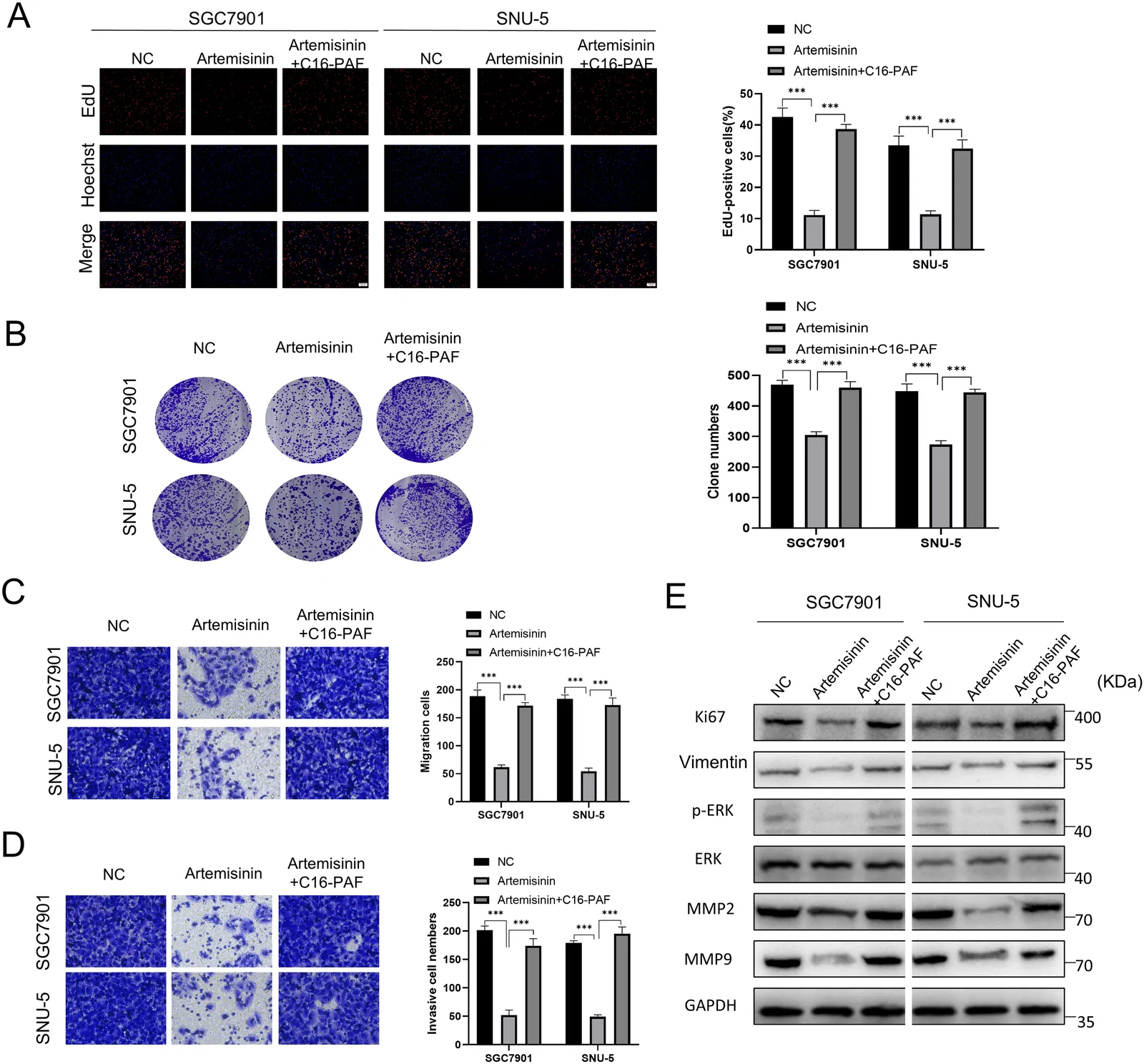
C16-PAF reverses artemisinin-induced inhibition of proliferation, migration, and invasion in gastric cancer cells. **(A)** EdU rescue assay. Representative fluorescence images and quantitative analysis of EdU-positive cell ratio. **(B)** Colony formation rescue assay. Representative images of colonies and quantitative analysis of colony numbers. **(C)** Transwell migration rescue assay. Representative images and quantitative analysis of migrated cell numbers. **(D)** Transwell invasion rescue assay. Representative images and quantitative analysis of invaded cell numbers. **(E)** Western blot analysis of p-ERK, Ki-67, Vimentin, MMP2, and MMP9 in SGC7901 and SNU-5 cells treated with artemisinin alone or in combination with C16-PAF. GAPDH served as the loading control. Control, artemisinin alone, and artemisinin + C16-PAF groups are shown. Data are presented as mean ± SD from three independent experiments. ***p < 0.001.

The Transwell assay results (**Figures 7C, D**) showed that the numbers of migrating and invading cells were significantly lower in the artemisinin-treated groups than in the control group. Following combined treatment with C16-PAF, the migration and invasion abilities were markedly restored, suggesting that the inhibitory effects of artemisinin on gastric cancer cell migration and invasion are also dependent on the inhibition of the ERK/MAPK pathway.

Western blot analysis (**Figure 7E**) showed that artemisinin treatment significantly reduced p-ERK levels in SGC7901 and SNU-5 cells, accompanied by markedly decreased expression of Ki-67, Vimentin, MMP2, and MMP9, while total ERK protein levels showed no significant change. Following the addition of C16-PAF, p-ERK levels recovered, and the expression of Ki-67, Vimentin, and MMP2/9 also returned to higher levels. Collectively, these results indicate that the ERK/MAPK signaling pathway is involved in mediating the inhibitory effects of artemisinin on gastric cancer cell proliferation, migration, and invasion, and that artemisinin treatment is accompanied by downregulation of *TOP2A*, *CCNB2*, and *CCNA2* expression.

## Discussion

The anti-gastric cancer activity of artemisinin has been reported in the literature (23); however, its specific targets and molecular mechanisms in gastric cancer remain unclear, which limits its further application in gastric cancer treatment. In this study, through network pharmacology, machine learning, molecular docking, and *in vitro* experiments, we systematically analyzed the potential molecular mechanisms of artemisinin against gastric cancer and identified *TOP2A*, *CCNB2*, and *CCNA2* as potential hub targets. Molecular docking suggested potential binding affinity between artemisinin and these targets. *In vitro* functional experiments further confirmed that artemisinin inhibits the proliferation, migration, and invasion of gastric cancer cells. Mechanistic studies showed that artemisinin treatment decreased ERK/MAPK pathway activity, accompanied by downregulation of the expression of the above target genes.

Regarding target screening strategies, traditional network pharmacology studies usually rely solely on topological parameters of the PPI network (e.g., degree, betweenness) to identify hub targets (24). Although this approach can identify key nodes in the network, it overlooks the actual discriminative ability of genes in disease states, as a highly connected gene in the network may not necessarily be a key driver of disease progression. To overcome this limitation, this study introduced machine learning algorithms (random forest, LASSO regression, and SVM-RFE) into the target screening process for artemisinin against gastric cancer for the first time, establishing a two-layer screening strategy of “network topology + feature selection.” The intersection screening strategy of the three algorithms effectively avoided false-positive or false-negative results that may arise from a single algorithm, as different algorithms are based on different mathematical principles: random forest excels at capturing nonlinear relationships (25), LASSO regression has advantages in sparse feature selection (26), and SVM-RFE performs robustly in high-dimensional data analysis (27). This strategy significantly improved the reliability of hub target screening and provides a referenceable multi-omics integration approach for studying the mechanisms of natural products.

The three hub targets identified in this study, *TOP2A*, *CCNB2*, and *CCNA2*, are all cell cycle regulatory molecules. *TOP2A* encodes DNA topoisomerase IIα, which plays a critical role in DNA replication, chromosome condensation, and sister chromatid separation, and its overexpression can lead to genomic instability and malignant tumor progression (28). *CCNA2* and *CCNB2* are cyclins A2 and B2, respectively, which serve as regulatory subunits of CDK kinases and regulate the transition from S phase to G2 phase and the G2/M phase transition, respectively (29). These three molecules functionally form a continuous regulatory axis: the *CCNA2*-CDK2 complex initiates DNA replication (S phase), the *CCNB2*-CDK1 complex drives mitotic entry (G2/M phase), and *TOP2A* resolves DNA topological tension during mitosis to ensure proper chromosome segregation. In this study, all three targets were significantly highly expressed in gastric cancer tissues, and GSEA analysis showed that their high-expression groups were significantly enriched in cell cycle-related pathways such as E2F_TARGETS and G2M_CHECKPOINT, further supporting the hypothesis that they promote gastric cancer development and progression by accelerating cell cycle progression (30). Artemisinin may achieve multi-blockade of the gastric cancer cell cycle process by simultaneously interfering with these three coordinately working cell cycle regulatory nodes rather than targeting a single node.

Kaplan-Meier survival analysis revealed no significant association between the expression levels of these three genes and the overall survival of gastric cancer patients. Possible reasons include that cell cycle-related genes primarily regulate tumor proliferative activity and have limited direct impact on long-term patient prognosis (31). In addition, the clinical heterogeneity of gastric cancer patients in the TCGA database (e.g., different tumor stages, molecular subtypes, and treatment regimens) may obscure potential survival associations (32). Nevertheless, the consistently high expression of these targets in gastric cancer tissues still supports their feasibility as actionable targets for artemisinin intervention.

Molecular docking results showed that artemisinin exhibited low binding energies with all three hub targets (< -5.0 kcal/mol), a range comparable to previously reported docking results of artemisinin with other tumor-related targets (33). To further evaluate the reliability of these binding affinities, the docking results were compared with those of reference compounds reported in previous studies. For TOP2A, the binding energy of artemisinin (−6.83 kcal/mol) was comparable to that of the known TOP2A inhibitor etoposide (−6.645 kcal/mol) reported in a previous docking study using the TOP2A structure (PDB: 5GWK) and MOE software (34). Although the docking system and software differed from those used in our study, the comparable binding energies suggest that artemisinin has a binding affinity for TOP2A similar to that of a known TOP2A inhibitor. For CCNA2, since no specific inhibitor targeting the CCNA2 subunit alone is currently available, salicylic acid, a known CDK2/Cyclin A2-binding small molecule, was used as a reference; the binding energy of artemisinin with CCNA2 (−6.87 kcal/mol) was comparable to that of salicylic acid with Cyclin A2 (−6.8 kcal/mol) and the CDK2/Cyclin A2 complex (−6.1 kcal/mol) reported in a previous docking study using AutoDock Vina and the CDK2/Cyclin A2 structure (PDB: 1FIN) (35). For CCNB2, since no specific inhibitor targeting the CCNB2 subunit alone is currently available, the classical CDK1 inhibitor seliciclib was used as a reference; the binding energy of artemisinin with CCNB2 (−7.30 kcal/mol) was compared with that of seliciclib (−8.7 kcal/mol; PDB: 4Y72) reported in a previous docking study using AutoDock Vina (36). Although the binding energy of seliciclib was stronger than that of artemisinin, the value for artemisinin remained below −5.0 kcal/mol, suggesting potential binding affinity. Among the three hub targets, artemisinin had the lowest binding energy with CCNB2 (−7.30 kcal/mol), suggesting that CCNB2 may be the target with the highest theoretical binding affinity for artemisinin in the cell cycle regulatory network. Binding mode analysis revealed that artemisinin could form hydrogen bonds, hydrophobic interactions, and π-alkyl interactions with all three targets, and the diversity of these interactions provides a structural basis for the multi-target binding of artemisinin.

Molecular docking predicted that artemisinin has binding potential with the three targets, and *in vitro* experiments further validated its functional relevance. qRT-PCR results showed that artemisinin significantly downregulated the mRNA expression levels of the three targets, and functional experiments demonstrated that artemisinin inhibited the proliferation, migration, and invasion of gastric cancer cells. These results validated the network pharmacology predictions at both the transcriptional and functional levels.

The immune infiltration analysis revealed that the expression of TOP2A, CCNB2, and CCNA2 was negatively correlated with the infiltration of several immune cell types, particularly CD4+ T cells, CD8+ T cells, and macrophages. This inverse relationship suggests that high expression of these cell cycle-related genes may be associated with an immunosuppressive tumor microenvironment in gastric cancer. High expression of these genes may promote rapid tumor cell proliferation and lead to a relative decrease in immune cell infiltration within the tumor microenvironment. In addition, activation of the ERK/MAPK pathway, which is closely linked to these hub genes, is required for the transition of resting regulatory T cells into suppressive effector Tregs, thereby contributing to an immunosuppressive tumor microenvironment (37). Artemisinin has also been shown to modulate immune responses by reducing FOXP3+ regulatory T cells and myeloid-derived suppressor cells while increasing cytotoxic T cell infiltration in the tumor microenvironment (38), suggesting that its downregulation of these hub genes may indirectly influence the immune microenvironment. However, the causal relationship between these hub genes and immune cell infiltration requires further experimental validation.

The ERK/MAPK pathway is a core signaling axis regulating cell proliferation, differentiation, and survival, and is aberrantly activated in approximately 40%-60% of gastric cancer patients, which is generally associated with poorer clinical prognosis (39). In this study, artemisinin treatment significantly decreased p-ERK levels, while total ERK levels showed no significant change, indicating that artemisinin specifically inhibits ERK phosphorylation (40). Rescue experiments using the ERK pathway activator C16-PAF further confirmed that activation of the ERK pathway partially reversed the inhibitory effects of artemisinin on gastric cancer cell proliferation, migration, and invasion and simultaneously restored the expression levels of p-ERK and downstream proteins (Ki-67, Vimentin, MMP2, MMP9). The partial reversal effect of C16-PAF suggests that the ERK/MAPK pathway is one of the main pathways through which artemisinin exerts its effects, but other parallel pathways may also be involved, which requires further investigation. Furthermore, this study has not yet demonstrated whether the ERK/MAPK pathway directly regulates the transcriptional expression of *TOP2A*, *CCNB2*, and *CCNA2*. Moreover, whether artemisinin directly targets these proteins requires further biochemical experimental validation. Molecular docking is a theoretical prediction and does not directly demonstrate physical binding between artemisinin and the target proteins. To substantiate direct drug–protein interactions, biophysical binding assays such as Cellular Thermal Shift Assay (CETSA), Surface Plasmon Resonance (SPR), or Drug Affinity Responsive Target Stability (DARTS) should be performed in future studies.

Relatively high micromolar concentrations of artemisinin (150 μM for SGC7901 and 200 μM for SNU-5) were employed in our *in vitro* functional assays. As a natural product originally developed as an antimalarial agent, artemisinin generally requires high-micromolar exposure to produce measurable anti-tumor effects in gastric cancer cells, consistent with previous reports (22). Nevertheless, such high concentrations raise concerns regarding potential off-target effects. Artemisinin can be activated by Fe(II)-dependent cleavage of its endoperoxide bridge to generate reactive radicals that may oxidize cellular components and cause non-specific cytotoxicity (41). Consequently, the cellular phenotypes observed in this study may partly arise from such off-target actions rather than exclusively from modulation of the ERK/MAPK–cell-cycle axis.

Although relatively high concentrations of artemisinin (>100 μM) were used in this study and such concentrations are often considered non-specific, several lines of evidence support a targeted action rather than pure non-specific cytotoxicity, including specific downregulation of p-ERK without affecting total ERK, downregulation of TOP2A/CCNB2/CCNA2 mRNA, and partial reversal by the ERK activator C16-PAF. These findings suggest that, although high concentrations may cause some off-target effects, the anti-gastric cancer effects of artemisinin are at least partly mediated through the ERK/MAPK–cell-cycle axis.

Although the above findings have been obtained, several limitations still exist. First, the screening and validation of hub targets were mainly based on public databases and *in vitro* cellular experiments, lacking *in vivo* animal model validation. Therefore, the findings of this study are restricted to *in vitro* models, and mouse xenograft models are required before drawing translational claims regarding the anti-gastric cancer efficacy of artemisinin. The *in vivo* anti-gastric cancer effects of artemisinin and its regulatory effects on these three targets need to be further confirmed in xenograft tumor models. Second, this study validated the downregulation of mRNA levels of the three targets by artemisinin using qRT-PCR, but did not detect their protein expression levels. Subsequent studies are needed to further verify the regulatory effects of artemisinin on these target proteins. Third, molecular docking is a theoretical prediction, and we did not perform biophysical binding assays such as CETSA, SPR, DARTS, or ITC to directly demonstrate the physical binding of artemisinin to TOP2A, CCNB2, or CCNA2. Future studies using these methods are needed to confirm the direct binding.

## Conclusions

In this study, by integrating network pharmacology, machine learning, molecular docking, and *in vitro* experiments, we found that artemisinin inhibits ERK/MAPK signaling pathway activation and downregulates mRNA levels of cell cycle-related *TOP2A*, *CCNB2*, and *CCNA2*, which correlates with suppressed proliferation, migration and invasion of gastric cancer cells. These findings are restricted to *in vitro* models, and mouse xenograft models are required before drawing translational claims regarding the anti-gastric cancer efficacy of artemisinin. The direct causal regulation between ERK pathway and three hub genes requires further experimental validation. Molecular docking suggested potential binding affinity between artemisinin and the above targets. This study provides experimental and theoretical support for the application of artemisinin in gastric cancer treatment, and also demonstrates the feasibility of an integrated “network pharmacology–machine learning–experimental validation” strategy for investigating the mechanisms of natural products.

## Data Availability

The datasets used in this study are publicly available. Artemisinin-related targets were retrieved from the HERB (http://herb.ac.cn), PubChem (https://pubchem.ncbi.nlm.nih.gov/), Swiss Target Prediction (http://www.swisstargetprediction.ch/), and SuperPred (https://prediction.charite.de/) databases. Gastric cancer RNA-seq data were obtained from the UCSC Xena database (https://xena.ucsc.edu/), which integrates data from TCGA and GTEx. The STAD RNA-seq data are available from the TCGA database (https://portal.gdc.cancer.gov/). All data generated or analyzed during this study are included in this published article and its supplementary information files.
