# Supplementary material for: Exploring the potential mechanism of artemisinin against gastric cancer based on network pharmacology, machine learning and experimental validation
Source: Front Oncol. 2026 Oct 27;16:1931227. doi: 10.3389/fonc.2026.1931227 (PMC13747746; doi:10.3389/fonc.2026.1931227)

Supplementary Material

Supplementary Figure S1. Hierarchical clustering heatmap of samples before and after outlier removal.

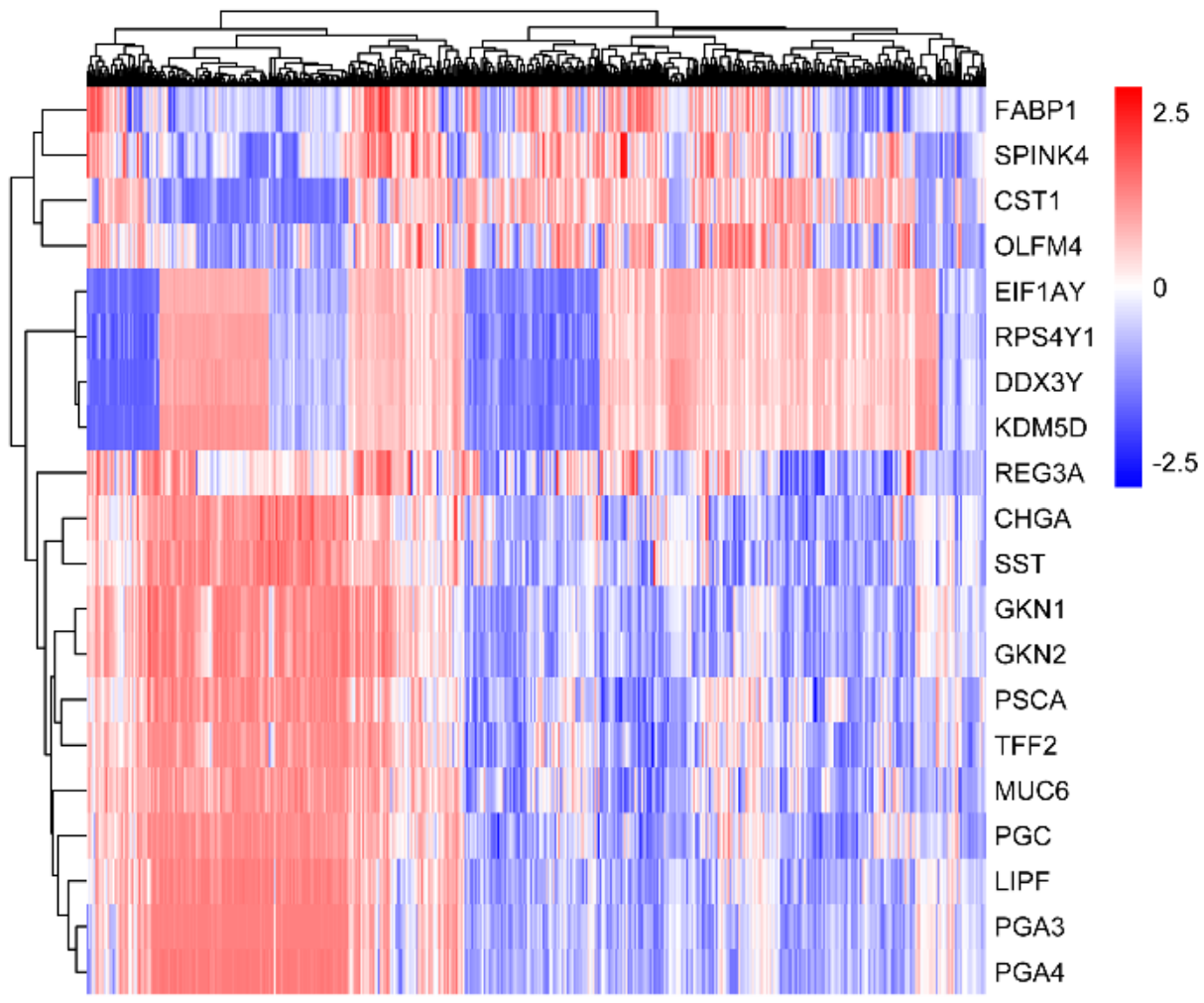

Supplementary Figure S2. WGCNA soft-thresholding power selection.

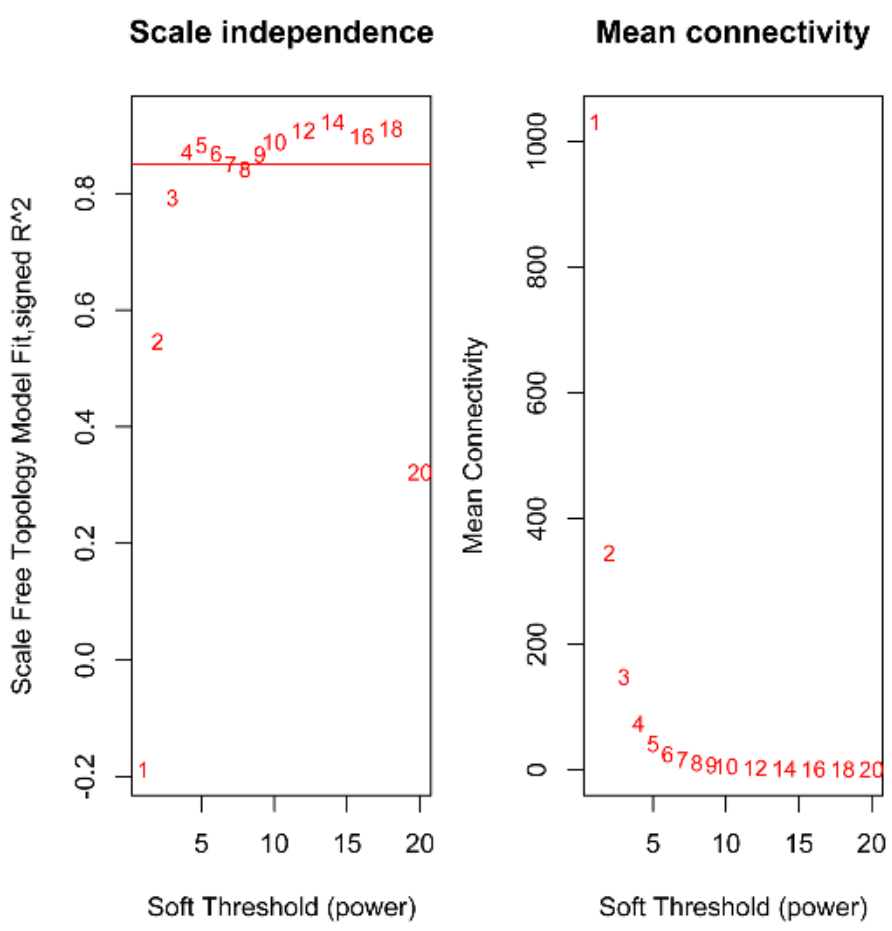

Supplementary Figure S3. Gene clustering dendrogram and module division in WGCNA. A total of 11 co-expression modules were identified by dynamic tree cutting.

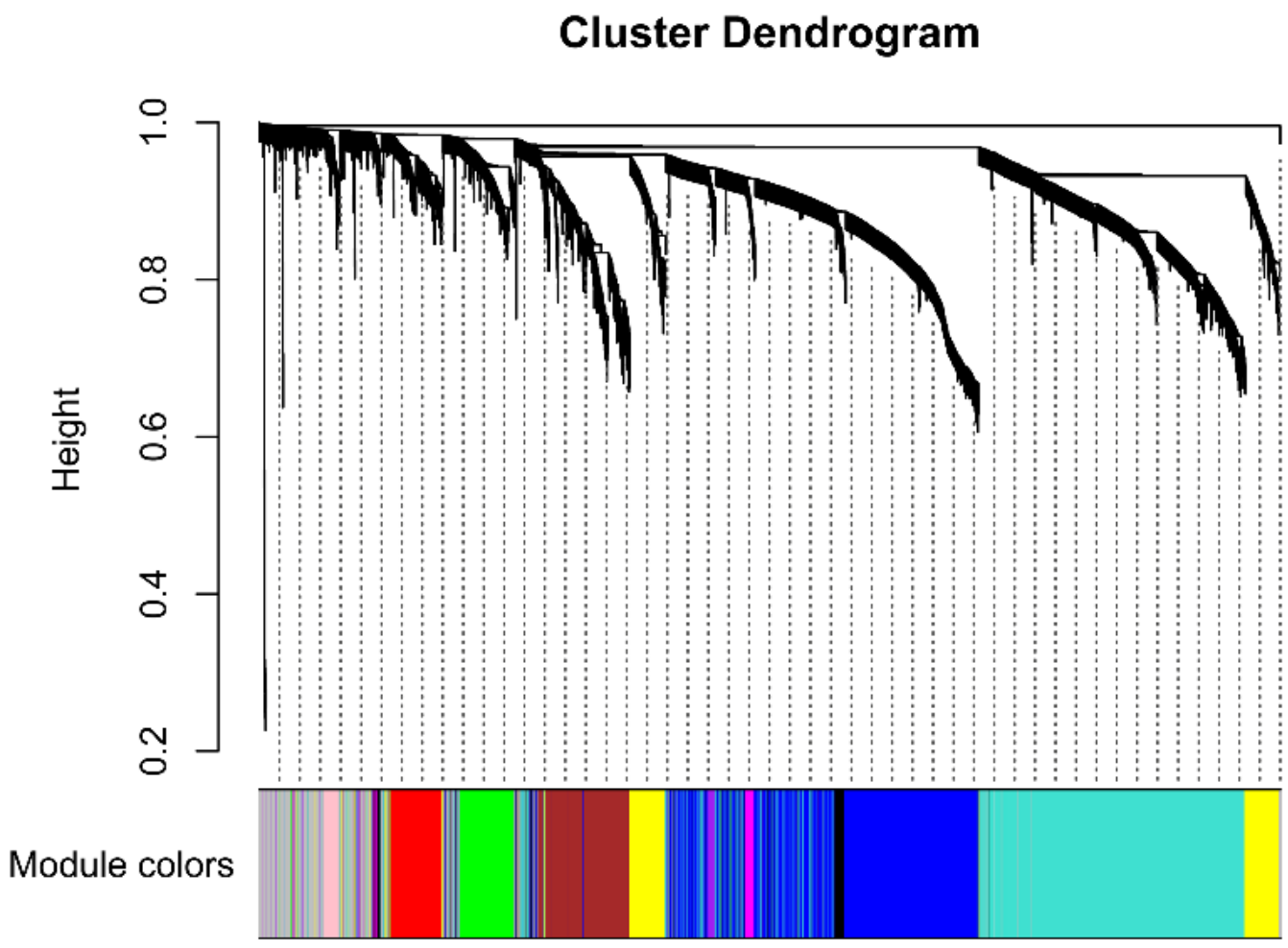

Supplementary Figure S4. Random forest model classification error as a function of the number of decision trees.

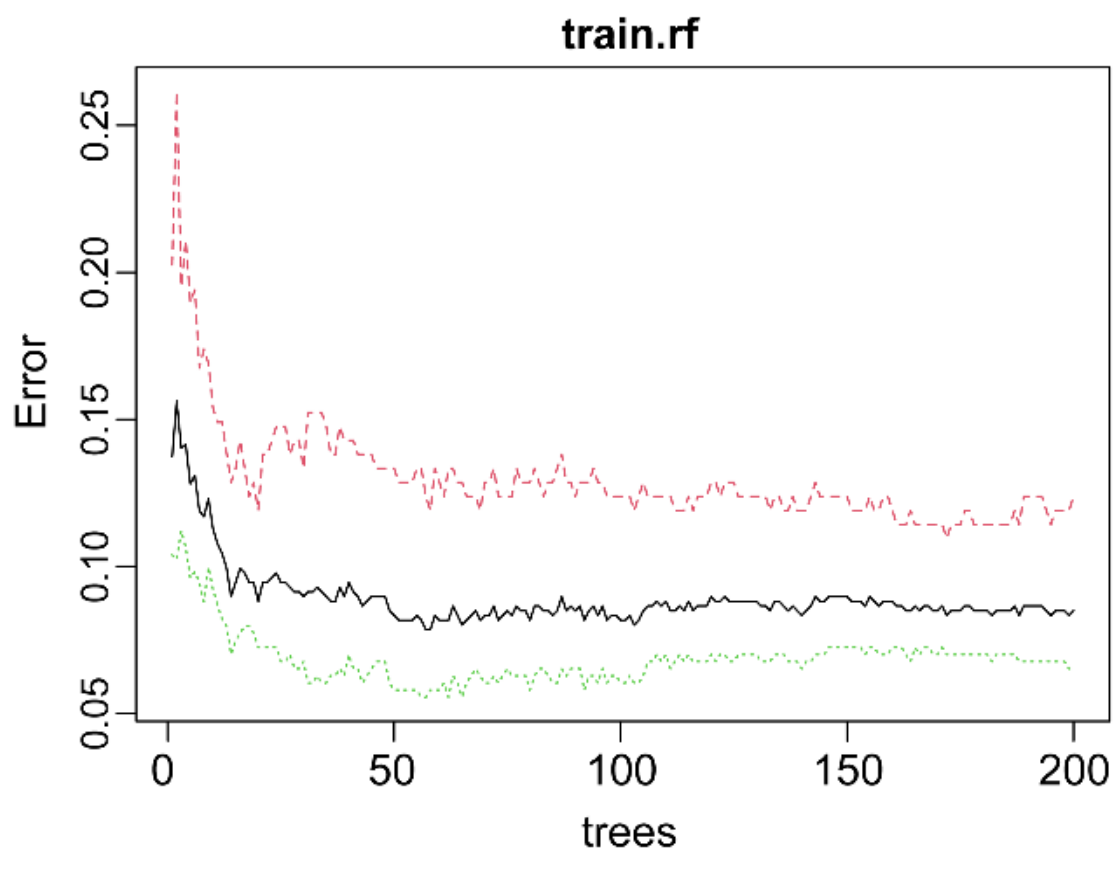

Supplementary Figure S5. Importance ranking of candidate hub targets based on the random forest model.

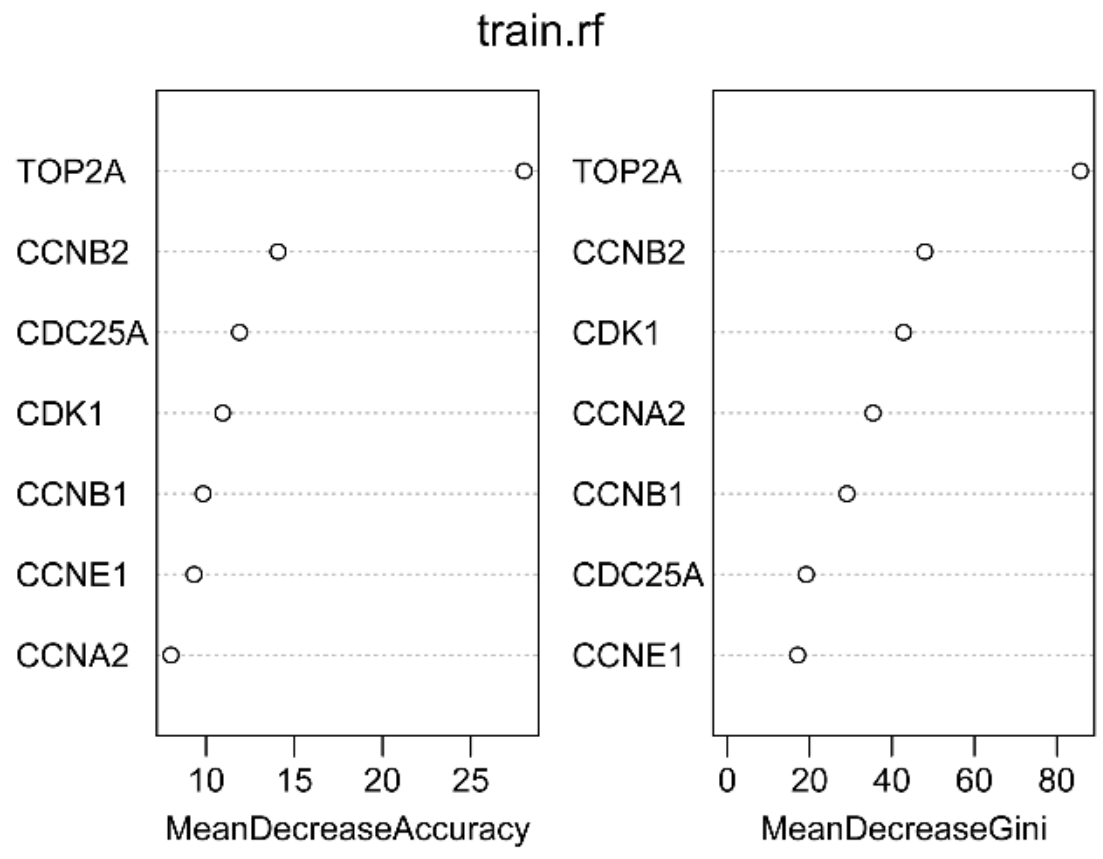

Supplementary Figure S6. LASSO regression coefficient profiles of candidate targets.

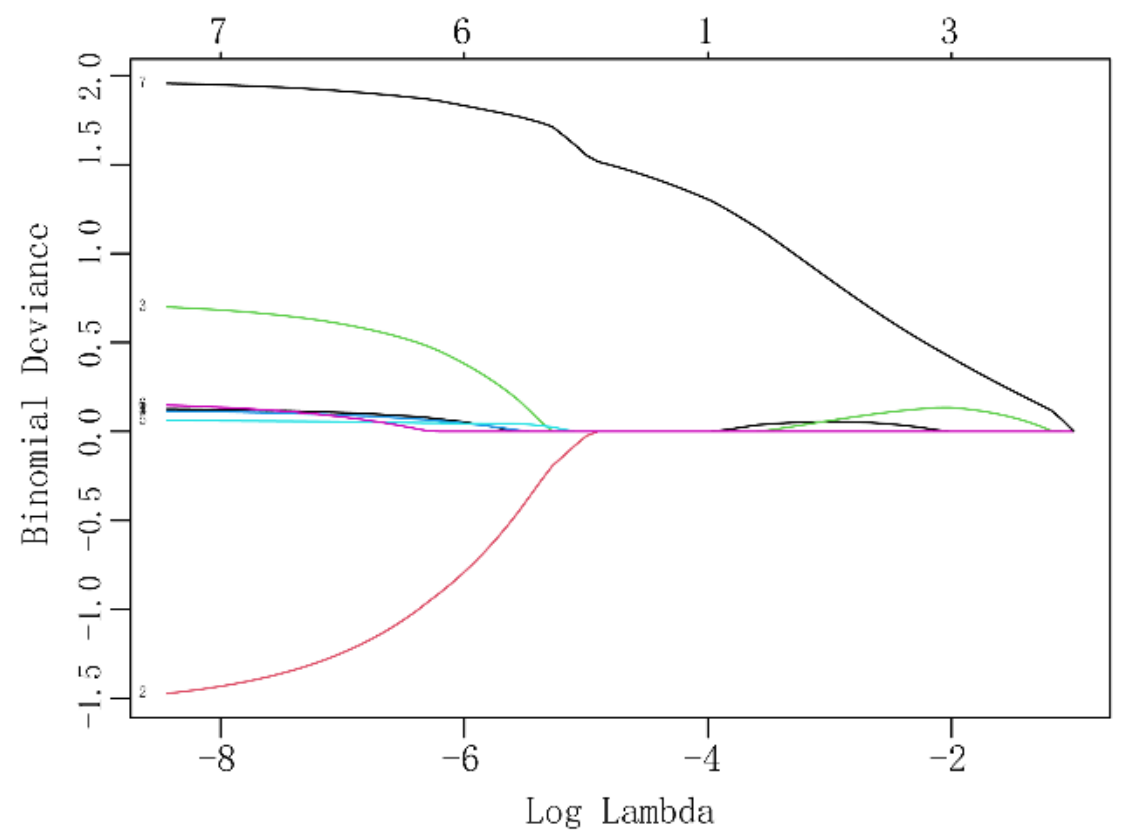

Supplementary Figure S7. Cross-validation curve for LASSO regression.

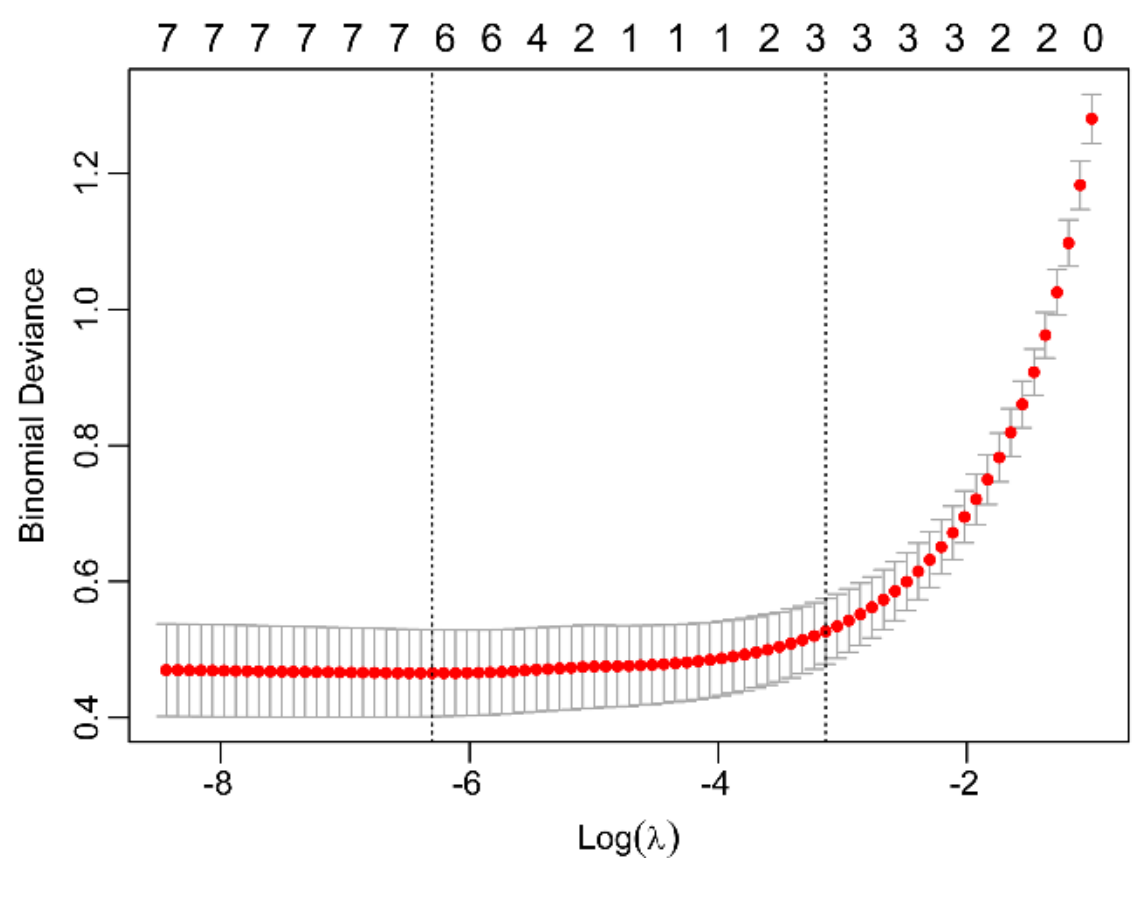

Supplementary Figure S8. SVM-RFE cross-validation error as a function of feature number.

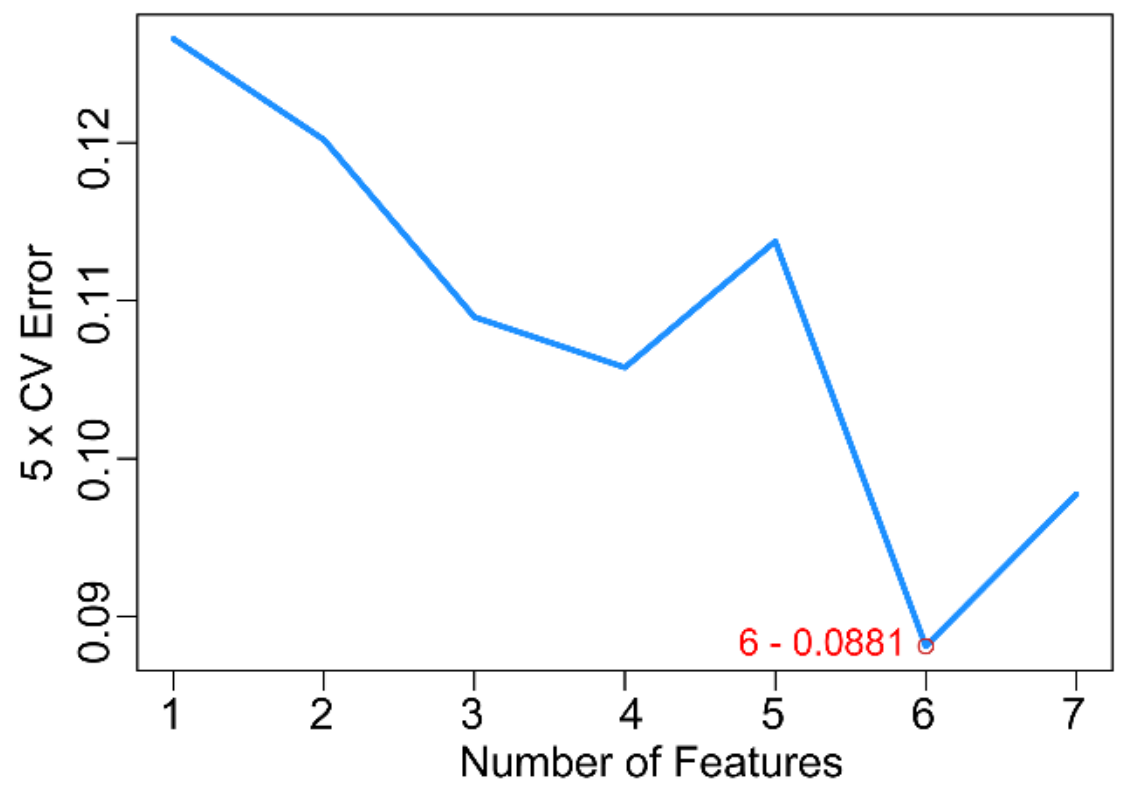

Supplementary Figure S9. SVM-RFE cross-validation accuracy as a function of feature number.

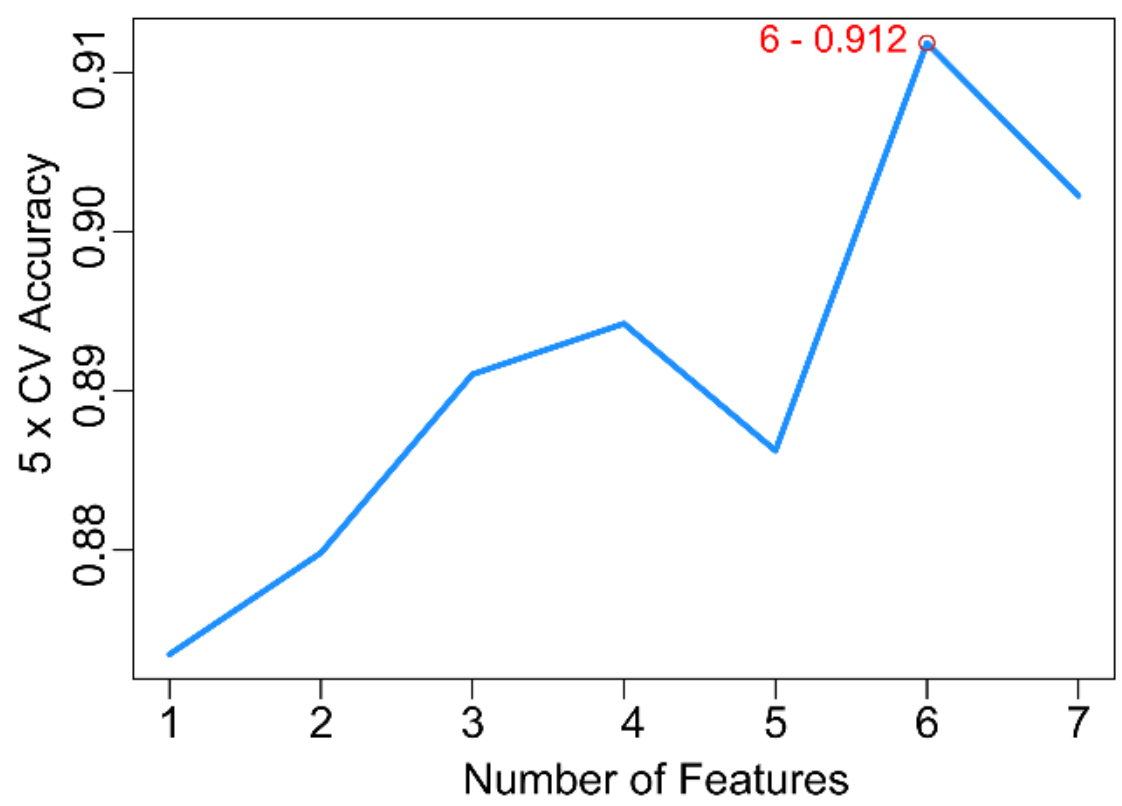

Supplementary Figure S10. Protein expression of TOP2A, CCNB2, and CCNA2 in gastric cancer tissues by immunohistochemistry (HPA database).

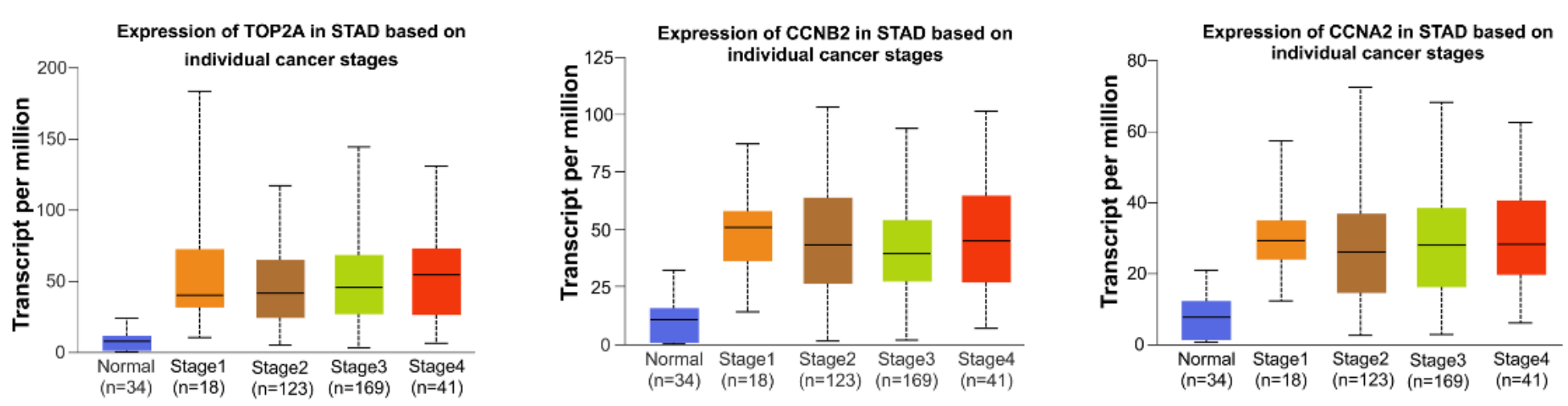

A B C

Supplementary Figure S11. Kaplan-Meier survival curves of TOP2A, CCNB2, and CCNA2 in gastric cancer patients.

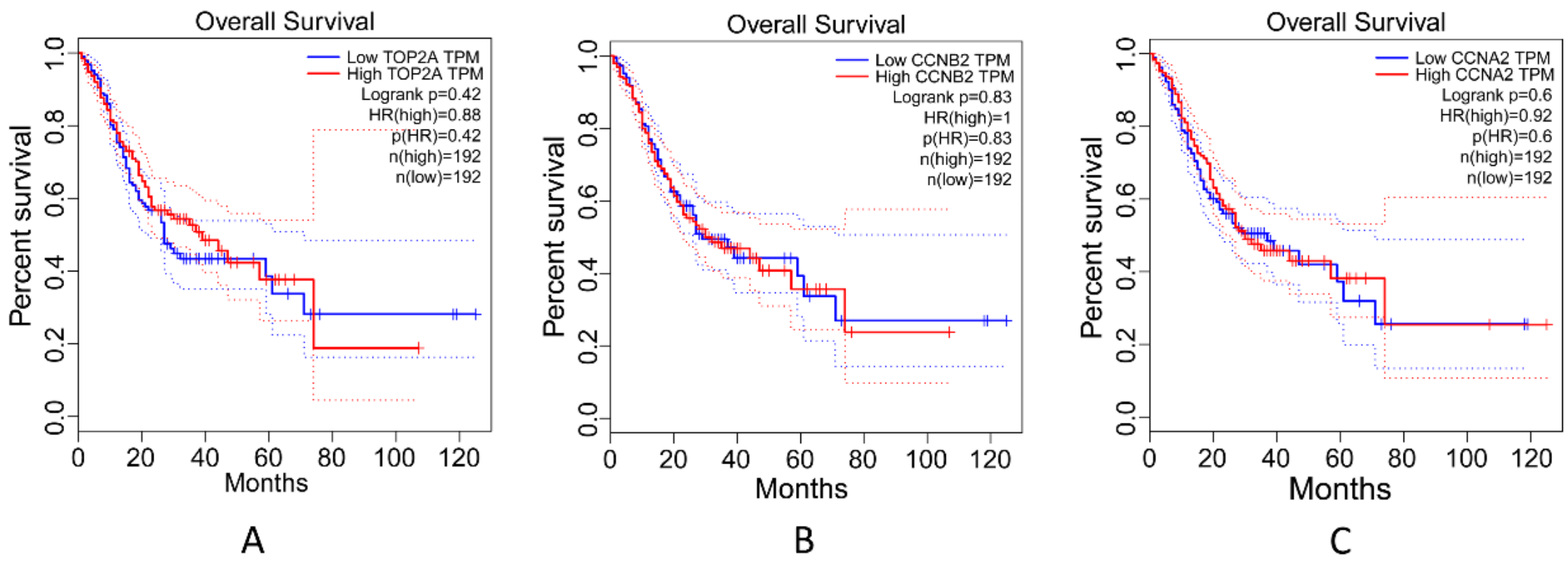

Supplementary Figure S12. Dose-response curves of artemisinin in SGC7901 and SNU-5 cells.

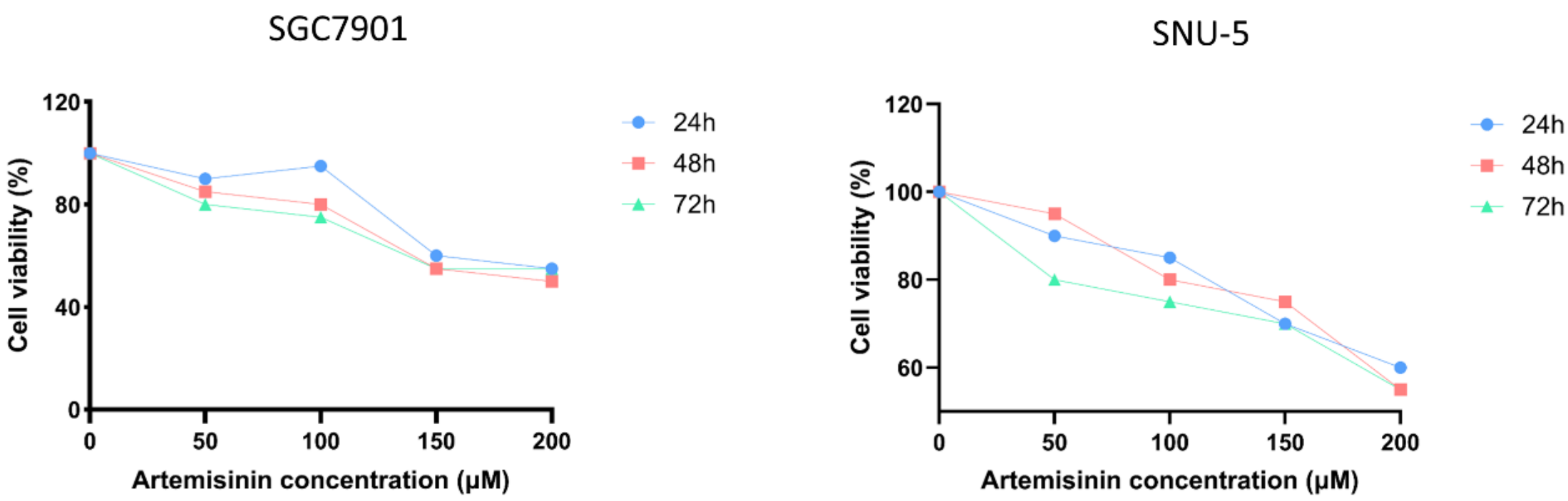

Supplement: Supplementary file 1 [file DataSheet1.pdf]
